# Simplified Spectrum Score (S^3^) app for pathogen-agnostic antimicrobial drug spectrum ranking to assess for antimicrobial de-escalation events

**DOI:** 10.1038/s41598-024-60041-6

**Published:** 2024-04-29

**Authors:** Mikaël de Lorenzi-Tognon, Jacques Schrenzel

**Affiliations:** 1 Bacteriology Laboratory, Division of Laboratory Medicine, Department of Diagnostics, Geneva University Hospitals, 4 Rue Gabrielle-Perret-Gentil, 1211 Geneva 14, Switzerland; 2 Genomic Research Laboratory, Faculty of Medicine, University of Geneva, Geneva, Switzerland; 3 Division of Infectious Diseases, Department of Medicine, Geneva University Hospitals, Geneva, Switzerland

**Keywords:** De-escalation, Antimicrobial stewardship, Antibiotics, Antimicrobial resistance, App, Ranking, Antibiotics, Computational models, Data integration, Data processing, Software, Infectious-disease epidemiology, Clinical microbiology, Bacterial infection

## Abstract

Antimicrobial/antibiotic de-escalation (ADE) is a key feature of antimicrobial stewardship programs (ASP) that relies mainly on individual panels for determining ADE events based on subjective ranking of antibiotics’ spectrum activity. The lack of consensus among ASP experts leads to reproducibility issues in the measure of this clinical outcome, making difficult to assess its real impact on patient care. The S^3^ score (Simplified Spectrum Score) app was developed to allow an objective ranking of antibiotics. Ranking was achieved by developing a database harboring pairs of bacteria-antibiotics for which each molecule was assigned a score based on published and clinically validated data from a recognized international committee. S^3^ score shows a strong correlation relationship and substantial agreement to a clinically validated spectrum score, and its framework enables any person to use it for ADE detection without assuming prior knowledge or training. In addition, its design enables regular updates and sustainability.

## Introduction

Antibiotics have been used worldwide since the discovery of penicillin in the years 1940’s and over the following years with the development of other classes of antibiotics. Unfortunately, bacteria have developed keen mechanisms to counter their actions and the prevalence of antibiotic resistance has been increasing ever since. It is now considered as a major public health concern that threatens the management of infectious diseases on a global scale. As of June 2023, the World Health Organization declared it a top priority research topic that must be answered by 2030 to tackle antimicrobial resistance worldwide^1^. One of the main drivers of antimicrobial resistance is the inappropriate use of antimicrobial drugs which has prompted the development of Antimicrobial Stewardship Programs (ASP).

ASPs aim at promoting the appropriate usage of antimicrobials to stall the selective pressure for emerging resistant pathogens. It is achieved by favoring drugs that satisfy the following conditions: the drug harbors the narrowest spectrum of activity on other bacterial species and is backed by evidence of successful clinical outcomes in patients. Appropriateness is usually judged on in vitro activity, and assessed through standardized antimicrobial susceptibility testing that defines thresholds for susceptible or resistant microbes such as the European Committee on Antimicrobial Susceptibility Testing (EUCAST) clinical breakpoints, and on their spectrum of activity on other microorganisms. Therefore, antimicrobial/antibiotic de-escalation (ADE) represents one way of assessing the efficacy of ASPs (quality indicator).

A generally accepted definition of ADE is the process of changing an initial broad-spectrum antimicrobial drug, which is active on a wide range of microorganisms, to a narrow-spectrum one that targets a smaller population of distinct microorganisms^2^. Ranking antibiotics’ spectrum of activity is crucial for detecting an ADE event. However, as of now, no consensus has been reached among experts, and there is no uniform antibiotic ranking system, leading to significant heterogeneity in the measure, not to mention the comparability, of this clinical outcome. This issue is likely the main reason why past studies failed to demonstrate any reduction on mortality in patients receiving this intervention^2^. ADE events are qualitative clinical outcomes that can be measured similarly to a diagnostic assay in laboratory medicine. One of the main drivers of analytical performance is precision, which corresponds to the measurement of independent replicates under the same experimental conditions^3^. In this context, precision is affected by the heterogeneity in ranking by antimicrobial stewardship experts who independently and subjectively assess the activity of each spectrum of antibiotics leading to an increased bias.

In order to limit imprecision and source of bias as well as to increase reproducibility, diverse attempts have been explored by researchers such as the development of spectrum scores^4–7^. One that has received much attention is the Madaras-Kelly et al*.* Spectrum Score which has been clinically validated and shows excellent performance at detecting ADE^7^. Nonetheless, it necessitates complex manual calculations that cannot be used by an untrained people and are subjects to errors. Moreover, it is still based on subjective criteria such as a weighted spectrum score for prone-to-resistance pathogens, as well as the credit score for an iv-oral switch^8^.

Our objectives were to develop a simple and user-friendly iOS application (app) that could be used without assuming prior knowledge or training to assess spectrum score metrics for ADE events detection. The S^3^ score (Simplified Spectrum Score) app enables an objective ranking of antibiotics, to standardize the measurements and decrease the heterogeneity introduced by panels of experts.

## Methods

### Database development of bacteria-antimicrobial drug pairs

**Figure 1:**
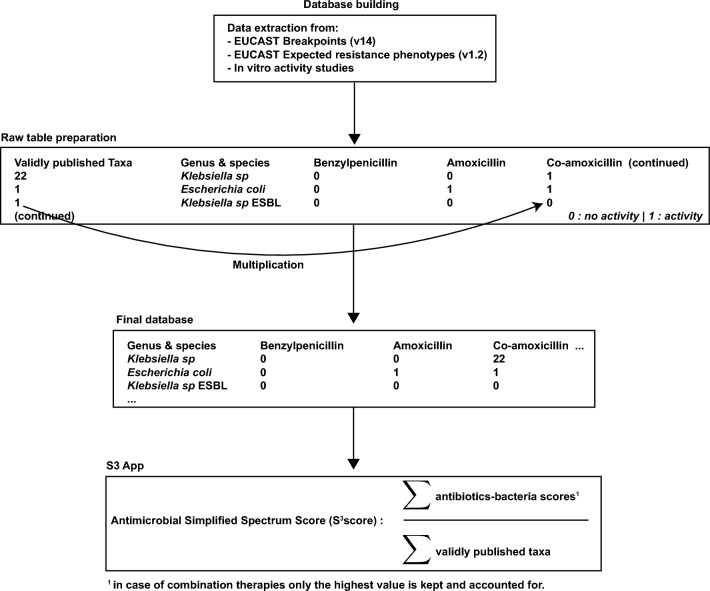
Database conception of the S^3^ score iOS^®^ App. Database building included extraction of data from EUCAST Breakpoints v14, EUCAST Expected Resistance Phenotypes v1.2 and selected in vitro activity studies (**A**). Raw table included antimicrobial drugs and their respective documented in vitro activity on all bacterial species of interest, taking into account the number of validly published taxa for each bacterium or row (**B**). Final database for spectrum score calculation which included the number of validly published taxa multiplied by the binary score (0: inactive/insufficient data, 1: active) for each row (**C**). Illustration of the final formula computing the S^3^ score stating that in case of combination therapy, only the highest value is retained for score calculation to avoid overlapping coverage of antimicrobial drugs (**D**).

The first step and the cornerstone of the S^3^ app was to build a database that incorporates the activity of a given antimicrobial drug for each of the bacteria of interest. The database included 111 bacteria as either unique bacterial species or resistant phenotypes (*e.g.,* carbapenemase-producing enterobacterales) which represent a broad set of pathogens that can be encountered in the clinical setting (Supplementary Figure S1). Antibiotics spectrum ranking was achieved by building matrices of bacteria-drug, the drug activity was coded as a binary outcome (0 or 1) and was assessed for each of the 111 bacteria represented in the database. More specifically, the first column of the database included all bacteria of interest, whereas the first row included all antimicrobial drugs of interest. For each bacterium-drug couple where a score of 0 (no in vitro activity or insufficient data) or 1 (evidence of in vitro activity) was assigned (Fig. 1), this number was then multiplied by the number of taxonomic units in order to achieve taxa normalization (see below). Only bacteria with validly published taxonomic units were included according to the list of prokaryotic names with standing nomenclature^9^.

### Antimicrobial S^3^score metrics assignment

In an effort of standardization, EUCAST clinical breakpoints^10^ and Expected Resistant Phenotypes were used to assign scores^11^. Data was extracted from the latest version of each file, namely, EUCAST clinical breakpoints v14 and Expected Resistant Phenotypes v1.2, both publicly and freely available at EUCAST’s website. In case a clinical breakpoint was missing, a review of the literature was performed to assess the in vitro activity of the missing values. These situations mainly concerned atypical bacteria such as *Legionella pneumophila*, *Chlamydia* species and other infrequent or difficult-to-culture bacterial species. The latest information available (December 2023) on in vitro susceptibility using similar methodology as EUCAST, either E-test or disk-diffusion testing, was used to populate the database^12–23^.

### Antimicrobial drugs used in combination

Antimicrobial drugs were considered as monotherapy except for aminoglycosides in Gram-positive bacterial species, which are mainly administered in combination to a beta-lactam. In case of combination of antimicrobial drugs, only the maximal value was considered to compute the final S^3^ score to avoid a falsely high spectrum score due to overlapping coverage of bacterial species. The theoretical maximal spectrum score value is 100%.

### Mitigating the potential sources of biases with taxa normalization

The main goal of S^3^ score was to estimate the activity of an antimicrobial drug on a wide range of bacterial species to rank them, however mass spectrometry cannot always identify bacteria to the species level and some species might be included in complexes, which can lead to over/under reporting of bacterial species in the database and be a source of bias. For instance, amoxicillin-clavulanate harbors an activity on anaerobic bacterial species such as *Bacteroides* whose genus includes 51 validly published species^9^. In this context, not considering the number of species in the *Bacteroides* genus would lead to a lower-than-expected spectrum score. S^3^ score was developed to mitigate this risk of bias, through the normalization of spectrum scores using the number of validly published taxa for bacterial species. The number of validly published species was multiplied to the initial dichotomic spectrum score (0 = inactive, 1 = active) to calculate the weighted value. As an illustration, the pair *Bacteroides*-amoxicillin-clavulanate would be assigned an individual score of 51 (Fig. 1). Ultimately, all paired bacteria-antimicrobial drugs scores were aggregated and divided by the total number of taxa for normalization, which represents 837 unique validly published taxonomical units for the 111 bacteria included in our database. For instance, oxacillin and flucloxacillin are only active against methicillin-susceptible *Staphylococcus* species and some streptococci, these represent 5 occurrences in our database, namely the methicillin-susceptible staphylococci group (validly published taxa = 12), *Staphylococcus capitis* (validly published taxa = 1), *Staphylococcus saprophyticus* (validly published taxa = 1), *Staphylococcus aureus* (validly published taxa = 3) and streptococci from Lancefield groups A, B, C and G (validly published taxa = 7). The dichotomic drug activity value (0 = inactive, 1 = active) assigned for these bacteria and flucloxacillin was then multiplied by their respective number of validly published taxa (n = 12 × 1 + 1 × 1 + 1 × 1 + 3 × 1 + 7 × 1 + 813 × 0 = 24). The final spectrum score was generated by dividing this number (n = 24) by the total number of validly published taxa included in the database (n = 837), to obtain the S^3^ score, 24/837 = 0.0286 or 2.86%.

### Antibiotics activity spectrum ranking and local prevalence of resistance

S^3^ score was developed to include the expected phenotypic resistance of bacterial species only^11^ in order to mitigate the risk of getting higher spectrum score for antimicrobial drugs with known narrower spectrum of activity, and in an effort for the app to be useful in different epidemiological settings. Local antimicrobial resistance epidemiology would affect spectrum scores and lead to falsely low or high scores depending on the local prevalence, supported by the potential inconsistency observed in Madaras-Kelly’s scoring system which shows a higher score for piperacillin-tazobactam compared to meropenem. These situations would be expected if one is using local epidemiological data from a high prevalence of resistant organisms to meropenem for instance. Rather, the S^3^ score was designed to be modular and adaptable to other antimicrobial stewardship tools such as the Desirability of Outcome Ranking for the Management of Antimicrobial Therapy (DOOR-MAT)^24^ system. DOOR-MAT integration enables to compute antimicrobial drug ranking with S^3^ score and desirability outcome based on the local prevalence of antimicrobial resistance. This system is adaptable to any geographic situation without having to change all spectrum scores in the database.

### S^3 ^quality controls and precision

Similarly, to any quantitative clinical assays, we followed the Clinical Laboratory Improvement Advisory (CLIA) recommendation and generated coefficient of variation based on quality controls ADE vignettes that we developed. Any database update is expected to affect the precision of S^3^ either because new bacterial species or new clinical breakpoints are added. To measure this effect on the delta score, which is used as a proxy to detect ADE, we calculated the S^3^ scores using our set of hypothetical clinical vignettes (n = 10) illustrating 5 situations of antimicrobial de-escalation and 5 other which would constitute an escalation [Supplementary Table S1). As EUCAST clinical breakpoints are updated roughly once a year, we calculated S^3^ scores of antimicrobial drugs using the previous database version (v13.1, now deprecated) and v14.0 (current) to generate a coefficient of variation (CV) (Supplementary Table S2). An acceptable range of the S^3^ delta score (ΔS^3^) is illustrated in Supplementary Table S2, using a CV < 30% as a precision metrics. Similarly to any quantitative clinical assay, any result falling outside of this range should prompt investigation about a potential issue with either the database (after an update for instance) or the algorithm itself.

### Statistical analyses

Accuracy was assessed by comparing its ability to detect ADE events using clinical vignettes published by Madaras-Kelly and using their scoring system as a gold standard^7^. The latest version of the revised Madaras-Kelly spectrum score^8^ was used to generate the scores for each antimicrobial regimen listed in the clinical vignettes (Table 1). Likewise, S^3^ scores were calculated for each antimicrobial regimen within their respective clinical vignette. An ADE was defined as a negative spectrum score delta by subtracting the final spectrum score value to the initial one. Quantitative analysis was performed by plotting normalized Madaras-Kelly against S^3^ scores delta scores to calculate Spearman’s correlation coefficient. Qualitative analysis was performed by using Madaras-Kelly’s spectrum score as a comparator method for ADE events, which helped building 2 × 2 tables to assess agreement between the two methods. All antibiotic’s spectrum scores individually available are reported in [Supplementary Table S2, Supplementary Figure S2) in a color-coded fashion. Statistical analyses were performed using Python version 3.12.0 with pandas (v2.1.3), numpy (v1.26.2), matplotlib (v3.8.2), seaborn (v0.13.0), pygal (v3.0.4) and scipy (v1.11.4).

**Table 1:** Clinical vignettes of antimicrobial de-escalation (ADE) events as published by Madaras-Kelly et al*.*^7^ with reported spectrum scores from Madaras-Kelly and S^3^ scoring systems.

Vignette ID	Initial (empirical) therapy	Final (targeted) therapy	Initial S^3^ score	Final S^3^ score	ΔS^3^	Initial Madaras-Kelly score	Final Madaras-Kelly score	ΔMadaras-Kelly	Ouctome
1	vancomycin piperacillin-tazobactam	ertapenem	93.07	49.22	− 43.85	84.51	63.32	− 21.19	ADE
2	vancomycin piperacillin-tazobactam levofloxacin	vancomycin imipenem	96.54	92.11	− 4.43	98.64	81.25	− 17.39	ADE
3	moxifloxacin	ceftriaxone	42.89	34.29	− 8.60	62.77	55.16	− 7.61	ADE
4	ceftriaxone azithromycin	levofloxacin	38.47	62.72	24.25	68.21	78.53	10.32	NDE
5	cefepime linezolid	ceftaroline	80.17	11.11	− 69.06	77.99	43.48	− 34.51	ADE
6	vancomycin piperacillin-tazobactam	vancomycin piperacillin-tazobactam levofloxacin	93.07	96.54	3.47	84.51	98.64	14.13	NDE
7	ciprofloxacin ampicillin-sulbactam	ciprofloxacin amoxicillin-clavulanate	94.62	95.22	0.60	84.24	84.24	0.00	NDE
8	piperacillin-tazobactam	ampicillin-sulbactam	58.78	28.08	− 30.70	76.63	43.75	− 32.88	ADE
9	vancomycin	trimethoprim-sulfamethoxazole	42.53	43.49	0.96	19.57	57.07	37.50	NDE
10	vancomycin piperacillin-tazobactam	moxifloxacin clindamycin	93.07	67.74	− 25.33	84.51	66.03	− 18.48	ADE
11	ceftazidime gentamicin	gentamicin imipenem	48.15	94.62	46.47	70.38	79.08	8.70	NDE
12	imipenem	moxifloxacin	89.84	42.89	− 46.95	73.10	62.77	− 10.33	ADE
13	ceftriaxone	piperacillin-tazobactam	34.29	58.78	24.49	50.75	76.63	25.88	NDE
14	tigecycline	ertapenem	23.30	49.22	25.92	82.61	63.32	− 19.29	ADE (MK) NDE (S^3^)
15	clindamycin	vancomycin	52.57	42.53	− 10.04	11.68	19.57	7.89	NDE (MK) ADE (S^3^)
16	vancomycin piperacillin-tazobactam	levofloxacin piperacillin-tazobactam	93.07	77.30	− 15.77	84.51	91.85	7.34	NDE (MK) ADE (S^3^)
17	levofloxacin	moxifloxacin	62.72	42.89	− 19.83	78.53	62.77	-15.76	ADE
18	ceftriaxone azithromycin	cefpodoxime doxycycline	38.47	58.66	20.19	68.21	77.99	9.78	NDE
19	vancomycin piperacillin-tazobactam	piperacillin-tazobactam metronidazole	93.07	59.02	− 34.05	84.51	76.63	− 7.88	ADE
20	ciprofloxacin	levofloxacin	78.49	62.72	− 15.77	78.53	78.53	0.00	NDE (MK) ADE (S^3^)

*ADE* antimicrobial de-escalation event, *NDE* non-de-escalation event, *MK* Madaras-Kelly et al.^8^ spectrum score.

### S^3 ^source code

Source code of the S^3^ score application is freely available in open source at GitHub (https://github.com/metg1985/S3score).

## Results

### General use of S^3 ^as a simple and user-friendly app to assess for ADE

**Figure 2:**
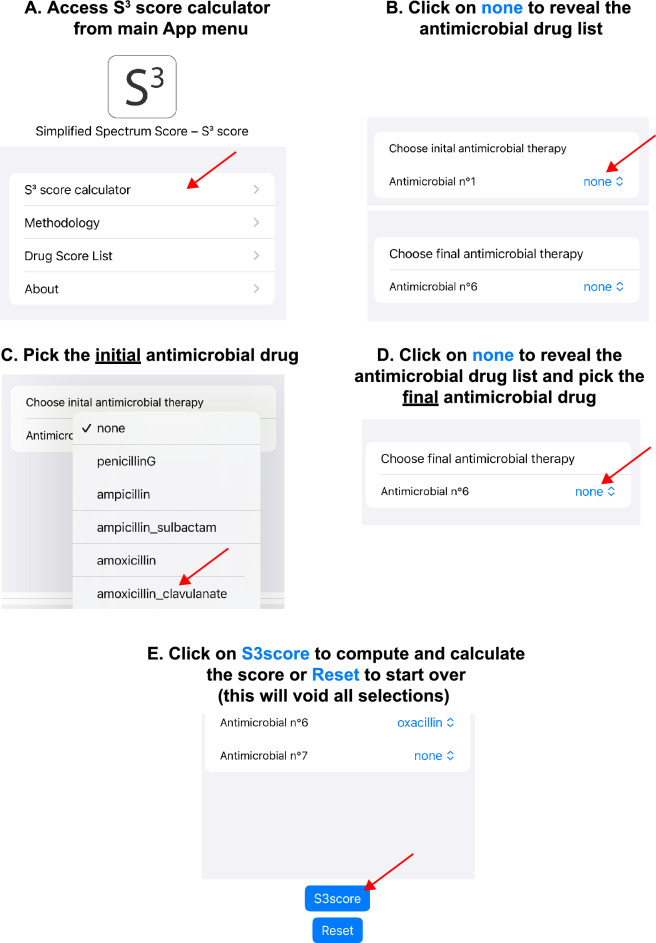
General user interface of the S^3^ app depicting the main menu of the app (**A**), clicking on the S^3^ score calculator enabled the user to reach the data input menu (**B**) where initial antimicrobial drugs can be chosen from a list (**C**), after the first choice is made the app will automatically propose a subsequent antimicrobial drug as input. After the user has finalized the final antimicrobial drug inputs (**D**), he can click on “S3score” button to assess for a de-escalation (ADE) event (**E**). The app will automatically compute the difference between final (targeted) and initial (empirical) therapy and display the resulting ΔS^3^ score.

**Figure 3:**
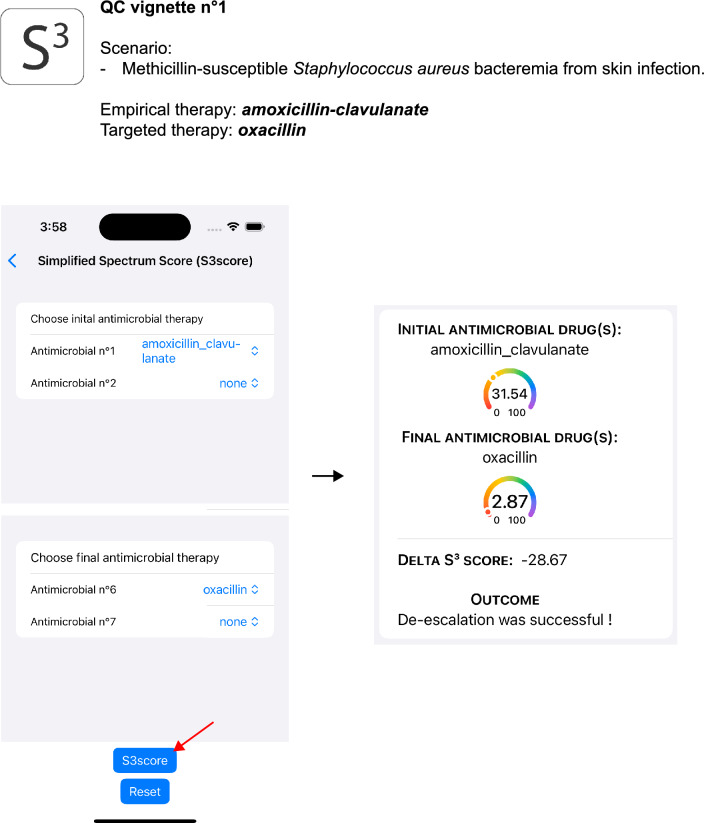
Example of a clinical situation when S^3^ can be used. This example is based on the Quality Control (QC) vignettes developed by the authors. In this scenario, a skin infection to *Staphylococcus aureus* leading to a bloodstream infection (bacteremia) and empirically treated (initial antimicrobial therapy) with amoxicillin-clavulanate. Once the antimicrobial susceptibility testing (AST) results are available, the clinician alters the empirical therapy by stopping amoxicillin-clavulanate and introducing oxacillin (*i.e.,* targeted or final therapy). When this data is fed into the app, the delta (ΔS^3^) score is displayed with an interpretation. Interpretation of the delta (ΔS^3^) score is given according to the following rule: ADE (ΔS^3^ < 0) or non-de-escalation (NDE, ΔS^3^ ≥ 0).

Figure 2 depicts the user-interface of the S^3^ app and a basic walkthrough to input the data required for the calculation of the spectrum scores. These inputs correspond to the initial antimicrobial therapy, or drugs from which the switch is made, and the final antimicrobial or the resulting drug after the switch is made. Quality controls ADE vignettes were developed to ensure and check for any underlying issue with S^3^. They provide examples of scenarios on how and when to use S^3^ to assess for ADE (Fig. 3, Supplementary Figure S3 and Supplementary Figure S4). For instance, Fig. 3 depicts a patient suffering from a bacterial skin infection caused by methicillin-susceptible *S. aureus* with bacteremia. In this hypothetical case, the initial (or empirical) therapy was amoxicillin-clavulanate. Subsequently, once the data on antimicrobial susceptibility is available, amoxicillin-clavulanate is stopped and substituted by oxacillin, a staphylococcal-specific synthetic penicillin. Since there are no other antimicrobial used in this scenario, no other input data is required to proceed with the calculation of the delta score. Clicking on the “S3score” button brings the user to another page displaying the delta S^3^ score or ΔS^3^ = − 28.67% (defined as final antimicrobial therapy S^3^ score minus initial antimicrobial therapy S^3^ score). Since the delta S^3^ score is negative (ΔS^3^ < 0), the outcome describes an antimicrobial de-escalation (ADE).

### Precision of S^3 ^and effect of database updates

Similarly to any quantitative clinical assays, we developed QC metrics to ensure the reliability of S^3^ following database and/or core code updates. Following the database update from v13.1 to v14.0 of EUCAST clinical breakpoints, we were able to confirm that the precision fell under the acceptable range, defined as a coefficient of variation not greater than 30% (Supplementary Table S2). The coefficient of variation is defined as the standard deviation divided by the average of observed/calculated values and is a key metric when assessing precision in clinical quantitative assays^25^. Unsurprisingly, the database updates, containing additional clinical breakpoints, especially for new or recent antimicrobial drugs, represent the outliers (CV > 30%). However, database updates should not breach the threshold of CV30% for antimicrobial drugs that harbor already a large amount of evidence on activity spectrum. We propose to use this data to benchmark any update of S^3^ and publish it, also encouraging any potential user to do the same to ensure reproducibility.

### S^3^ score shows a strong correlation to the Madaras-Kelly spectrum score

**Figure 4:**
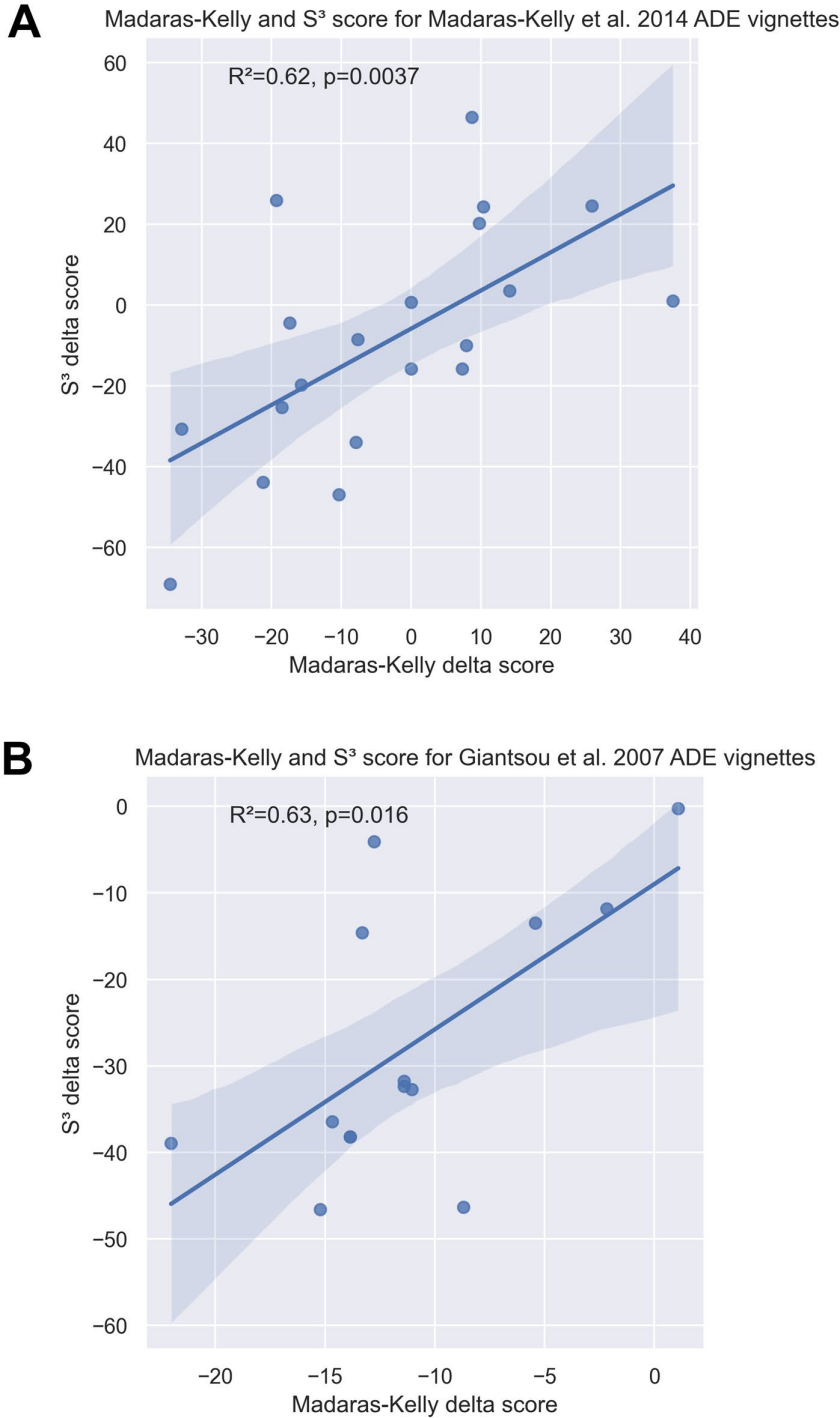
Quantitative comparative analysis of Madaras-Kelly and S^3^ scoring systems using clinical antimicrobial de-escalation (ADE) vignettes developed by Madaras-Kelly et al.^7^ (**A**), and using the clinical ADE cases depicted in Giantsou et al.^26^ (**B**). The plot shows a linear regression with the corresponding R^2^ score and p-value for statistical significance.

The first step to assess S^3^ score accuracy was to compare the delta scores, i.e. the difference between the final spectrum score (or targeted therapy) and the initial (or empirical therapy), of S^3^ and Madaras-Kelly scores using the latter as the gold standard (Table 1). Overall, S^3^ score metrics show a strong correlation relationship to the Madaras-Kelly scores (Fig. 4A) (Spearman coefficient = 0.62). Moreover, agreement between the two metric systems was substantial when assessing only the qualitative outcome ADE or NDE (no de-escalation) with positive, negative and overall percent agreement of 90.0% [95%CI: 59.6%–98.2%], 70.0% [95%CI: 39.7%–89.2%] and 80.0% [95%CI: 58.4%–91.9%], respectively. However, discordant results were observed in four clinical vignettes (vignette 14–15-16–20).

### Discordant delta spectrum scores are caused by imbalances in relative proportion of bacterial species

Interestingly, half of the discordant results included vancomycin and were evaluated by the Madaras-Kelly scoring system as no-de-escalation events (vignettes 15–16, Table 1). Vancomycin is a glycopeptide exclusively active against Gram-positive bacteria, which account only for 19% of all bacterial species included in Madaras-Kelly. The vancomycin spectrum score harbors an aggregate score of 16/92 = 17.4%, lower than S^3^ score = 42.5%. Since S^3^ score considers the relative proportion of all bacterial species, it was less affected by imbalances in the relative proportion of Gram-positive and Gram-negative bacteria, thus explaining the discordant results for clinical vignette 15 and 16. Likewise, tigecycline illustrated in clinical vignette 14 scored an aggregate of 76/92 = 82.6%, and 23.3% with the S^3^ score for the same reasons. The last case, vignette 20 suggests a better resolution in the antimicrobial spectrum score from S^3^ compared to Madaras-Kelly. Whereas levofloxacin and ciprofloxacin were assessed independently for their spectrum score in S^3^, Madaras-Kelly chose to consider them as identical.

### Comparative analysis in an independent cohort confirms strong relationship between S^3 ^and Madaras-Kelly in detecting ADE events

To compare the overall performance of both S^3^ and Madaras-Kelly in detecting ADE in an independent cohort of antimicrobial de-escalation event, a literature search was conducted to identify a study that included a set of clinical cases illustrating ADE events. The study needed to state in detail which antimicrobial drugs were used as an initial treatment regimen, as a final treatment regimen and the clinical outcome (de-escalation or not) to be selected, to be able to compute the spectrum delta scores using both scoring systems. A study satisfying all criteria was identified^26^ and used for the comparative analysis. Giantsou et al*.* describe two groups of patients: one with de-escalation events (n = 14) and the other one without (n = 17) that included a total of 143 patients. In the latter group, none of the antibiotics regimens were changed leading to non-de-escalation events (NDE). Since each scoring system compared the regimens by subtracting the final score to the initial one, both scoring systems showed perfect negative percent agreement (100%). Only the unique ADE/NDE scenarios were considered for the comparative analysis, in opposition to the total number of patients. Main differences between scoring systems were observed in the antibiotic de-escalation group of patients (Table 2). Overall, Madaras-Kelly and S^3^ delta scores showed a strong relationship (Fig. 4B) in detecting ADE events in this independent cohort of patients with strong agreement on the qualitative outcome (de-escalation/no-de-escalation) with positive, negative and overall percent agreement of 100.0% [95%CI: 83.8%–99.4%], 94.0% [95%CI: 74.2%–99.0%] and 96.8% [95%CI: 83.8%–99.4%], respectively. Madaras-Kelly showed one discordant case (n°7) which depicted a switch from linezolid, meropenem and amikacin regimen to linezolid, piperacillin-tazobactam and amikacin. This was not identified as an ADE by Madaras-Kelly as the scoring system assigns a higher score to piperacillin-tazobactam than meropenem.

**Table 2:** Clinical cases of antimicrobial de-escalation (ADE) events as published by Giantsou et al*.*^26^ with reported spectrum scores from Madaras-Kelly and S^3^ scoring systems.

Cases id	Initial (empirical) therapy	Final (targeted) therapy	Initial S^3^ score	Final S^3^ score	ΔS^3^	Initial Madaras-Kelly score	Final Madaras-Kelly score	ΔMadaras-Kelly	Outcome
1	linezolid meropenem amikacin	piperacillin-tazobactam	95.22	58.78	− 36.44	91.30	76.63	− 14.67	ADE
2	linezolid meropenem quinolone	piperacillin-tazobactam	97.73	58.78	− 38.95	98.64	76.63	-22.01	ADE
3	linezolid piperacillin-tazobactam amikacin	ceftazidime amikacin	94.98	48.39	− 46.59	92.39	77.17	− 15.22	ADE
4	linezolid piperacillin-tazobactam quinolone	linezolid ceftazidime quinolone	97.13	85.30	− 11.83	98.64	96.47	− 2.17	ADE
5	linezolid meropenem quinolone	ceftazidime quinolone	97.73	65.35	− 32.38	98.64	87.23	− 11.41	ADE
6	linezolid meropenem amikacin	piperacillin-tazobactam amikacin	95.22	62.49	− 32.73	91.30	80.25	− 11.05	ADE
7	linezolid meropenem amikacin	linezolid piperacillin-tazobactam amikacin	95.22	94.98	− 0.24	91.30	92.39	1.09	NDE (MK) ADE (S^3^)
8	linezolid piperacillin-tazobactam amikacin	linezolid ceftazidime amikacin	94.98	81.48	− 13.50	92.39	86.96	− 5.43	ADE
9	meropenem quinolone	piperacillin-tazobactam	97.01	58.78	− 38.23	90.49	76.63	− 13.86	ADE
10	meropenem quinolone	piperacillin-tazobactam	97.01	58.78	− 38.23	90.49	76.63	− 13.86	ADE
11	meropenem amikacin	meropenem	94.74	90.68	− 4.06	85.87	73.10	− 12.77	ADE
12	meropenem amikacin	ceftazidime amikacin	94.74	48.39	− 46.35	85.87	77.17	− 8.70	ADE
13	linezolid piperacillin-tazobactam quinolone	ceftazidime quinolone	97.13	65.35	− 31.78	98.64	87.23	− 11.41	ADE
14	piperacillin-tazobactam quinolone	quinolone	77.30	62.72	− 14.58	91.85	78.53	− 13.32	ADE

*ADE* antimicrobial de-escalation event, *NDE* non-de-escalation event, *MK* Madaras-Kelly et al.^8^ spectrum score.

### S^3^ score enhances DOOR-MAT system by providing an objective and reproducible ranking system of antimicrobial drugs

**Figure 5:**
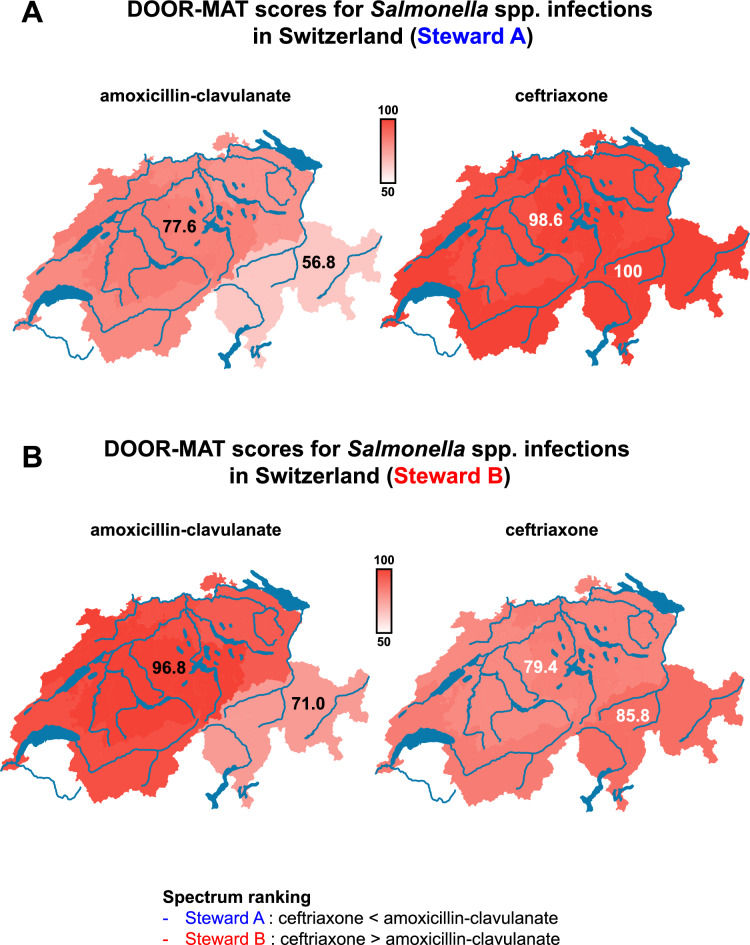
An example of S^3^ modularity through integration with the Desirability of Outcome Ranking for the Management of Antimicrobial Therapy (DOOR-MAT) tool is illustrated. A scenario involving the assessment of empirical treatment in *Salmonella spp.* infections in Switzerland is presented within the context of two antimicrobial stewards. Steward A ranks amoxicillin-clavulanate as having a broader spectrum of activity compared to ceftriaxone. The resulting DOOR-MAT scores are displayed for each region of Switzerland (**A**). Steward B ranks ceftriaxone as having a broader spectrum of activity in regard to amoxicillin-clavulanate. The resulting DOOR-MAT scores are displayed for each region of Switzerland (**B**). The higher the score, the greater the likelihood for an antimicrobial agent to be active against the microorganism based on local epidemiology. This scenario illustrates the impact of heterogeneity among antimicrobial stewardship experts and the lack of reproducibility of the outcomes it can lead to (*i.e.,* Steward A would conclude ceftriaxone is the best empirical therapy, whereas Steward B would come to the opposite conclusion).

S^3^ is a versatile and modular app that seamlessly integrates with existing antimicrobial stewardship tools. An example of this integration is demonstrated with DOOR-MAT^27^, short for Desirability of Outcome Ranking for the Management of Antimicrobial Therapy. Developed to assess antibiotic selection strategies within local guidelines, particularly to overcome drug resistance, DOOR-MAT employs a scoring system to gauge the desirability of outcomes. Higher scores signify more favorable outcomes, indicating the likelihood of empirical therapy with a narrow spectrum of activity effectively covering pathogens in specific regions with available antimicrobial susceptibility testing (AST) data. However, a notable concern with DOOR-MAT is its dependence on the user’s subjective perspective for ranking antimicrobial drug spectra, forming the foundation of its output metrics. S^3^ addresses this issue by incorporating natural and expected phenotypic bacterial resistances, rather than solely relying on local epidemiological AST data. This approach ensures the reproducibility of DOOR-MAT scores when conducting comparative analyses across different regions worldwide with varying levels of antimicrobial resistance. For example, discrepancies in ranking the spectrum of activity between ceftriaxone and amoxicillin-clavulanate, as highlighted by Weiss et al.^28^, can significantly impact DOOR-MAT scores. This discrepancy is evident in the differing susceptibilities of *Salmonella* species in Switzerland, with the Southern region exhibiting lower susceptibility to amoxicillin-clavulanate compared to ceftriaxone in the rest of the country. If Steward A, who ranks amoxicillin-clavulanate as having a broader spectrum of activity than ceftriaxone, were to use DOOR-MAT to evaluate Swiss guidelines on empirical treatment for *Salmonella* bloodstream infections, they would conclude that ceftriaxone should be the empirical treatment of choice (Fig. 5A, Supplementary Table S3). Conversely, if Steward B, who ranks ceftriaxone as having a broader spectrum of activity than amoxicillin-clavulanate, were to conduct the same analysis, they would not only reach an opposing conclusion but also have noticed the differences in antimicrobial resistance between South Switzerland and other regions (Fig. 5B, Supplementary Table S3). This example underscores the enhanced utility of both S^3^ and DOOR-MAT, offering additional layers of information essential to public health and antimicrobial stewardship.

## Discussion

Antimicrobial de-escalation (ADE) events pose challenges due to the lack of international consensus and effective metrics for ranking antimicrobial drug spectra. Existing studies have demonstrated inconsistency among antimicrobial stewardship (AMS) experts in their rankings, leading to imprecise clinical outcome measures. While Madaras-Kelly et al*.* have developed a clinically validated scoring system^7,8^, its reliance on manual calculations poses a risk of error and limits its practical use by clinicians and researchers. S^3^ represents a pioneering solution, offering a smartphone application for pathogen-agnostic and evidence-based ranking of antimicrobial drugs spectra in a user-friendly interface accessible to clinicians and researchers without specialized training. Utilizing publicly available Antimicrobial Susceptibility Testing (AST) data from EUCAST ensures reproducibility in ADE assessments. We have developed Quality Control (QC) metrics to monitor database and algorithm deviations, particularly during updates with new EUCAST clinical breakpoints released. Our study demonstrates S^3^’s reliability, showing strong correlation with the Madaras-Kelly et al. scoring system. Assessment of clinical ADE vignettes revealed almost perfect agreement between the two scoring systems in qualitative outcomes. Moreover, S^3^’s sustainability is evidenced by its capability to incorporate new data through database updates. In addition, S^3^’s modular design allows seamless integration with existing antimicrobial stewardship tools like DOOR-MAT, further enhancing their functionality.

Previous studies have emphasized the variability in the definition of ADE events, as demonstrated in a recent meta-analysis^29^. This analysis revealed that only a fraction (n = 4/14, 28.5%) provided explicit criteria for the ranking of antimicrobial agents, primarily conducted by antimicrobial stewards. However, these definitions exhibited heterogeneity and often categorized antimicrobials into classes, resulting in limited sensitivity to detect ADE. Similar flaws were observed in other studies^28,30–34^, mainly attributed to the subjective ranking of antimicrobial drug spectra by independent AMS experts. However, as highlighted earlier, the primary limitation associated with ADE is the current lack of standardization and homogeneity in ranking antimicrobial drug spectra, a challenge that S^3^ aims to address.

While ADE may offer a valuable metric for assessing the efficacy of ASPs compared to outcomes influenced by patient comorbidities and transfers between facilities, it is important to keep in mind limitations and pitfalls when using ADE as a measurable clinical outcome. De Waele et al*.*^35^ warn about using ADE as a quality index, as it may not accurately capture all the features of clinical cases of infection. This limitation arises when the empirical and the targeted therapies are identical, thus not resulting in an ADE event. The authors provide the following example: a *S. aureus* skin and soft tissue infection empirically treated with flucloxacillin, and unchanged after pathogen identification and antimicrobial susceptibility testing. Despite representing the best medical practice in a low antimicrobial resistance setting, this scenario would be penalized as it would not have been recorded as an ADE event. However, we believe a tool such as S^3^ might prove helpful to address these limitations as it quantifies any antimicrobial drug regimen. For instance, in a given cohort of patients, the incidence of ADE events identified by S^3^ can be compared to the distribution of initial (empirical) S^3^ score metrics. This could theoretically allow to assess the association between these two metrics and an outcome of interest, such as the incidence of antimicrobial resistance.

Another concern with empirical antimicrobial drugs that can affect ADE is the risk of an inactive treatment, which has been associated with longer length of stay and even higher mortality^36^. Subsequently, ADE becomes more likely when broad-spectrum antimicrobial therapies are used as empirical agents in regions with overall low levels of antimicrobial resistance (*e.g.,* switch from meropenem to ampicillin). Likewise, ADE prevalence may decrease in high levels of antimicrobial resistance (*e.g.,* empirical therapy with meropenem left unchanged when ESBL producers are detected with AST). This is why, in order to assess the empirical therapy harboring the highest likelihood of covering a pathogen in these regions, DOOR-MAT represents a perfect tool when integrated with S^3^ as illustrated in our study. This provides an additional layer of information to clinicians and researchers when local guidelines on empirical therapies must be assessed.

Several other scoring systems have been developed by researchers. One such system, cited in this study as a main comparator, is the scoring system developed by Madaras-Kelly et al. It has been proven to be a reliable tool for assessing ADE, demonstrating a sensitivity and specificity of 86.3% and 96.0%, respectively, against adjudication by three experienced antimicrobial stewards. However, the authors incorporated CLSI’s AST data beforehand to assign ordinal score values to organism-antimicrobial pairs. Additionally, they multiplied these scores by a factor of 1.25 or 1.75, depending on the organism, to reflect the potential for resistance development. These subjective choices influenced the final calculation of each antimicrobial spectrum score and led to one concerning inconsistency: a higher spectrum score for piperacillin-tazobactam compared to meropenem, which is contrary to the general literature^28^. Two other scoring systems, developed by Ilges et al.^4^ and Moehring et al.^5^, propose a similar pathogen-agnostic approach to rank antimicrobial drug spectra. The former is based on the Antimicrobial Spectrum Index (ASI) developed by Gerber et al.^6^, with slight modifications. Although ASI aligns with S^3^ in not relying on local epidemiological AST data, it includes a smaller panel of bacterial species (n = 15) as representative categories and lacks taxa normalization, increasing the risk of imbalances in the choice of representative bacterial species. Furthermore, ASI still requires manual calculations, unlike S^3^, making it less accessible to clinicians and researchers. Moehring et al. propose a simpler classification for antimicrobial drug spectra ranking, consisting of a 4-ordinal scoring system. This system is easier to remember and does not require complex manual calculations to assess for ADE events. It also includes a class of protected antimicrobial drugs for antimicrobial stewardship purposes. However, antimicrobial drugs included in each of the 4-ordinal categories exhibit overlapping S^3^ scores, suggesting that while practical, the scoring system may lack sensitivity to detect all ADE events due to its low resolution.

This study has several limitations. Firstly, the app incorporates subjective features such as assigning a default value of 0 to molecules with insufficient evidence of in vitro activity. This primarily applies to newly developed antimicrobial drugs like ceftobiprole, ceftaroline or dalbavancin/oritavancin. Secondly, carriers of extended-spectrum beta-lactamase, carbapenemase, or other antimicrobial resistance mechanisms were accounted for in the database. They were assigned a taxonomic unit value of 1, representing an overall frequency of 0.12% in the database but accounting for up to 80% for a single taxon (*e.g., Escherichia coli*), potentially introducing bias. However, we justify this choice as it balances the spectrum scores of all antimicrobial molecules and does not seem to affect the overall performance of the S^3^ score in detecting ADE events. Thirdly, local epidemiology was not considered in building the database, as the app was designed for broad usage in different epidemiological settings with varying levels of antimicrobial resistance. This issue is mitigated by the modularity of S^3^, which readily integrates with existing antimicrobial stewardship tools such as DOOR-MAT. Fourthly, relying on in vitro AST data has known limitations. Minimal inhibitory concentrations, the mainstay metrics of AST, can sometimes be influenced by growth culture media^37^. However, these cases are infrequent, and hospitals and clinicians still rely on AST to tailor their choice of therapy. Lastly, Madaras-Kelly’s scoring system, used as comparator in this study, represents an imperfect gold standard in detecting ADE events. Nonetheless, it should be noted that there is currently no gold standard for ADE event detection, and this comparator has been clinically validated by the authors, justifying its use as a comparator in our perspective.

In summary, S^3^ score framework enables any person to use it for ADE detection without prior knowledge or training. Its database developed on published and up-to-date clinical breakpoints increases reproducibility and limits potential biases on antimicrobial drug ranking. The app framework also allows for swift updates of the database upon release of new clinical breakpoint data. Taken together, S^3^ score app could improve the measurement and benchmarking of clinical outcomes in ADE studies and its modularity enables it to integrate perfectly to existing antimicrobial stewardship tools such as DOOR-MAT, enhancing them by enabling reproducibility via standardization. Nonetheless, clinical studies to confirm and validate the use of S^3^ will be needed to ensure its broad clinical utility, as the continuing effort into developing antimicrobial stewardship tools to further enhance precision and objectivity of assessing ASP outcomes.

### Supplementary Information

Supplementary Legends.

Supplementary Figure S1.

Supplementary Figure S2.

Supplementary Figure S3.

Supplementary Figure S4.

Supplementary Table S1.

Supplementary Table S2.

Supplementary Table S3.

## Data Availability

The authors confirm that the data supporting the findings of this study are available within the article. The S^3^ score app is freely available on the Apple Store^®^ for iOS devices. Source code of the S^3^ score application is freely available in open source at GitHub (https://github.com/metg1985/S3score). Raw data is available from the corresponding author (MDT) upon reasonable request.
