# Supplementary material for: Simplified Spectrum Score (S3) app for pathogen-agnostic antimicrobial drug spectrum ranking to assess for antimicrobial de-escalation events
Source: Sci Rep. 2024 Apr 29;14:9776. doi: 10.1038/s41598-024-60041-6 (PMC11059348; doi:10.1038/s41598-024-60041-6)
Supplement: Supplementary file 1 — Supplementary Legends. [file 41598_2024_60041_MOESM1_ESM.docx]

**SUPPLEMENTARY TABLES AND FIGURES LEGENDS**

**Supplementary Figure S1.** Heatmap matrix of antimicrobial drugs-bacteria pairs with color-coding (blue: inactive or insufficient data, black: active) illustrating the *in vitro* activity of antimicrobials on all the bacterial species included in the database (n = 111 bacteria for a total of n = 837 taxonomic units or taxa).

**Supplementary Figure S2.** Heatmap illustrating the S^3^ score in a color-coded fashion (black: broader spectrum or higher S^3^ value, blue: narrower spectrum or lower S^3^ value).

**Supplementary Figure S3.** Example of a clinical situation when S^3^ can be used. This example is based on the Quality Control (QC) vignettes developed by the authors. In this scenario, a bacterial meningitis caused by *Neisseria meningitidis* is treated empirically (initial antimicrobial therapy) with ampicillin and ceftriaxone. Once the microorganism is identified and its antimicrobial susceptibility testing (AST) results are available, the clinician alters the empirical therapy by stopping ampicillin and continuing ceftriaxone alone (*i.e.,* targeted or final therapy). When this data is fed into the app, the delta (ΔS^3^) score is displayed with an interpretation. Interpretation of the delta (ΔS^3^) score is given according to the following rule: ADE (ΔS^3^ < 0) or non-de-escalation (NDE, ΔS^3^ ≥ 0).

**Supplementary Figure S4.** Example of a clinical situation when S^3^ can be used. This example is based on the Quality Control (QC) vignettes developed by the authors. In this scenario, a severe urinary tract infection caused by extended-spectrum beta-lactamase producer *Escherichia coli* is treated empirically (initial antimicrobial therapy) with piperacillin-tazobactam. Once the microorganism is identified and its antimicrobial susceptibility testing (AST) results are available, the clinician alters the empirical therapy by stopping piperacillin-tazobactam and introducing meropenem alone (*i.e.,* targeted or final therapy). When this data is fed into the app, the delta (ΔS^3^) score is displayed with an interpretation. Interpretation of the delta (ΔS^3^) score is given according to the following rule: ADE (ΔS^3^ < 0) or non-de-escalation (NDE, ΔS^3^ ≥ 0).

**Supplementary Table S1.** Examples of antimicrobial de-escalation (ADE) events for which the acceptable delta S3 score (ΔS^3^) was calculated based on the latest database update (EUCAST clinical breakpoints v14.0, January 2024). The range reported accounts for a maximal value of 30% in the coefficient of variation.

**Supplementary Table S2.** Precision data based on the recent update of the database with new clinical breakpoints released by EUCAST on January 2024 (v14.0). All antimicrobial drugs available as input to S^3^ are displayed with their corresponding spectrum score value according to the version of EUCAST clinical breakpoints used (v13.1 or v14.0). The S^3^ score average between the two versions of the database and its standard deviation (std) is shown, allowing to calculate the coefficient of variation (CV) depicted in the last column.

**Supplementary Table S3.** Raw data of Desirability of Outcome Ranking for the Management of Antimicrobial Therapy (DOOR-MAT) matrices used to generate Figure 5 based on a scenario of *Salmonella* species infection in regions of Switzerland showing varying levels of antimicrobial susceptibilities to amoxicillin-clavulanate and ceftriaxone.
