# Supplementary figures and images for: Simplified Spectrum Score (S3) app for pathogen-agnostic antimicrobial drug spectrum ranking to assess for antimicrobial de-escalation events
Source: Sci Rep. 2024 Apr 29;14:9776. doi: 10.1038/s41598-024-60041-6 (PMC11059348; doi:10.1038/s41598-024-60041-6)

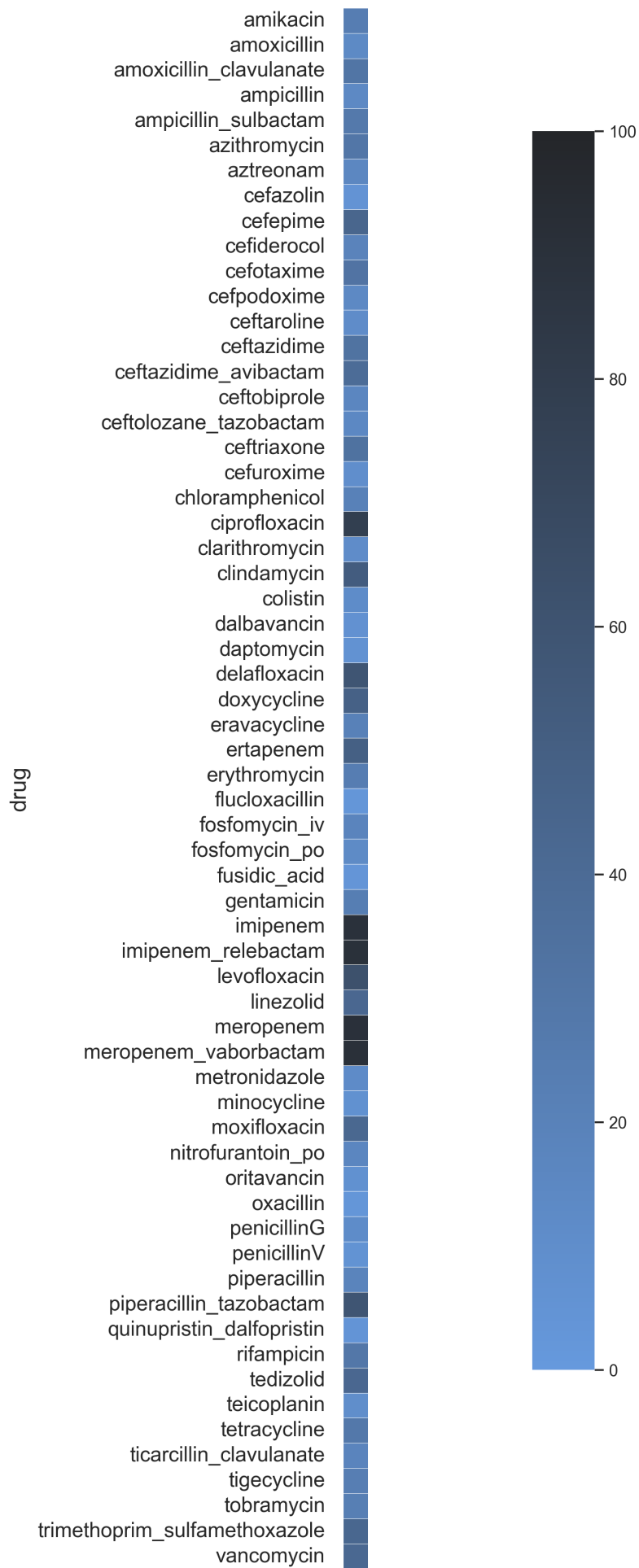

S<sup>3</sup> score

Supplement: Supplementary file 3 — Supplementary Figure S2. [file 41598_2024_60041_MOESM3_ESM.pdf]
