# Supplementary material for: Simplified Spectrum Score (S3) app for pathogen-agnostic antimicrobial drug spectrum ranking to assess for antimicrobial de-escalation events
Source: Sci Rep. 2024 Apr 29;14:9776. doi: 10.1038/s41598-024-60041-6 (PMC11059348; doi:10.1038/s41598-024-60041-6)

QC vignette n°4

Scenario:
- Bacterial meningitis caused by *Neisseria meningitidis*.

Empirical therapy: ***ampicillin + ceftriaxone***
Targeted therapy: ***ceftriaxone***

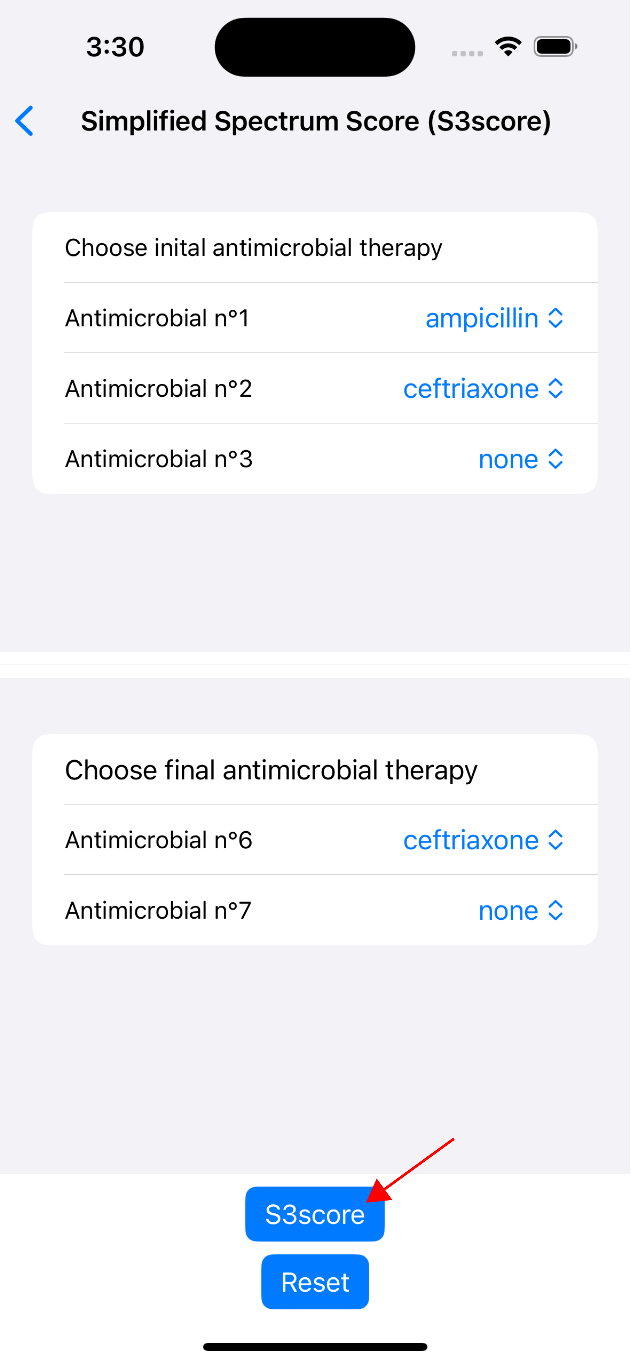

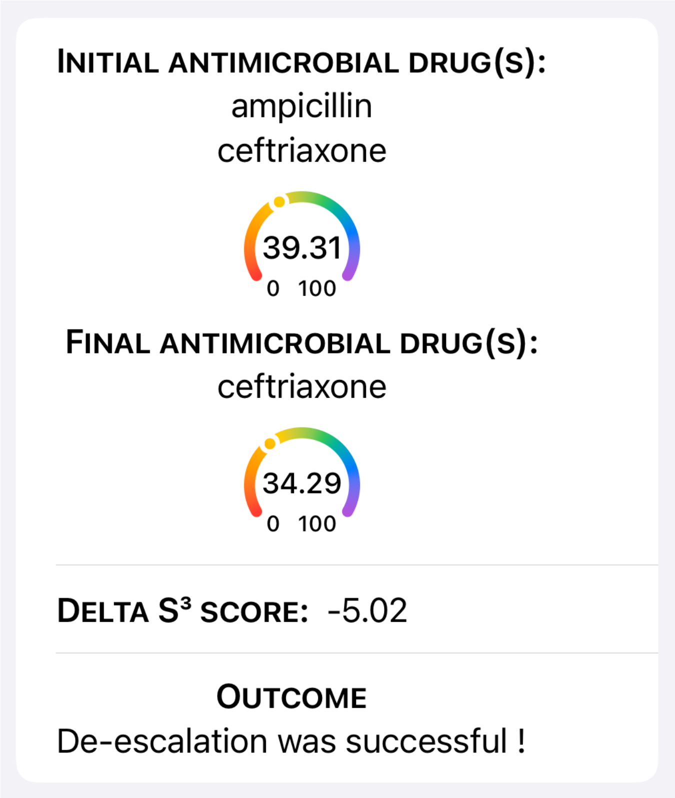

Supplement: Supplementary file 4 — Supplementary Figure S3. [file 41598_2024_60041_MOESM4_ESM.pdf]
