# Supplementary material for: Simplified Spectrum Score (S3) app for pathogen-agnostic antimicrobial drug spectrum ranking to assess for antimicrobial de-escalation events
Source: Sci Rep. 2024 Apr 29;14:9776. doi: 10.1038/s41598-024-60041-6 (PMC11059348; doi:10.1038/s41598-024-60041-6)

QC vignette n°6

Scenario:

- Severe urinary tract infection caused by *Escherichia coli* ESBL

Empirical therapy: ***piperacillin-tazobactam***
Targeted therapy: ***meropenem***

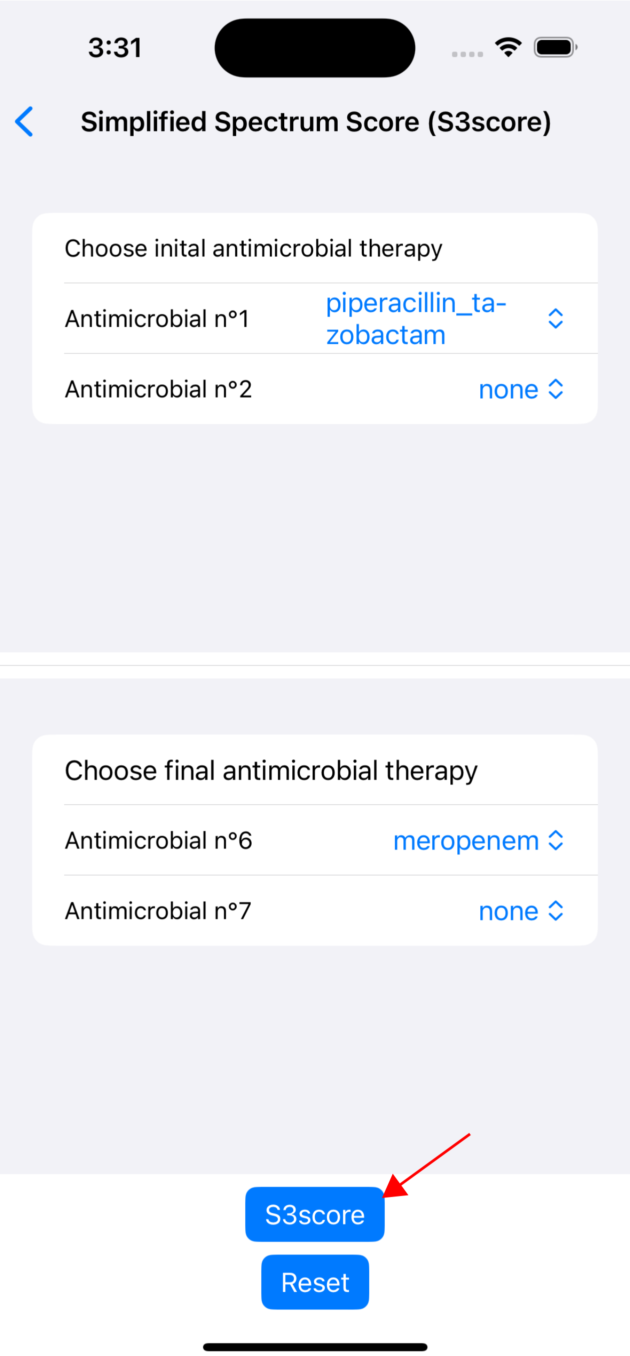

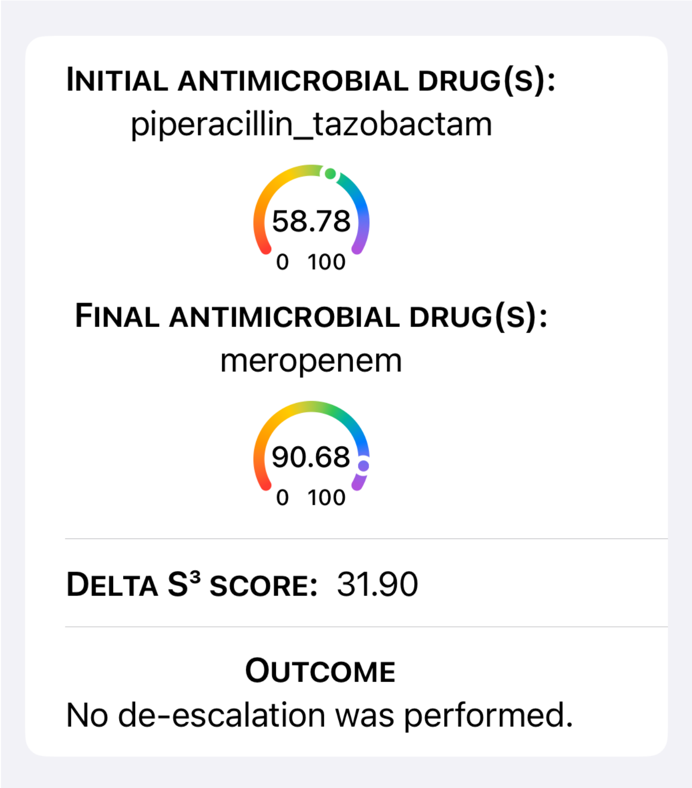

Supplement: Supplementary file 5 — Supplementary Figure S4. [file 41598_2024_60041_MOESM5_ESM.pdf]
