# Supplementary material for: Simplified Spectrum Score (S3) app for pathogen-agnostic antimicrobial drug spectrum ranking to assess for antimicrobial de-escalation events
Source: Sci Rep. 2024 Apr 29;14:9776. doi: 10.1038/s41598-024-60041-6 (PMC11059348; doi:10.1038/s41598-024-60041-6)
Supplement: Supplementary file 6 — Supplementary Table S1. [file 41598_2024_60041_MOESM6_ESM.docx]

**Supplementary Table X. Quality controls ADE vignettes for S^3^ score**

| Vignette ID | Description | Acceptable ΔS^3^ range (CV30%) |
| --- | --- | --- |
| 1 | Methicillin-susceptible *Staphylococcus aureus* bacteremia from skin infection, empirical therapy with co-amoxicillin, targeted therapy with oxacillin. | from -37.27 to -20.07 |
| 2 | *Streptococcus pneumoniae* community-acquired pneumonia, empirical therapy with ceftriaxone, targeted therapy with ampicillin. | from -26.72 to -14.39 |
| 3 | *Klebsiella pneumoniae* severe urinary tract infection with sepsis, empirical therapy with piperacillin-tazobactam, targeted therapy with co-amoxicillin. | from -35.41 to -19.07 |
| 4 | *Neisseria meninigitidis* bacterial meningitis, empirical therapy with ampicillin and ceftriaxone, targeted therapy with ceftriaxone. | from -6.53 to -3.51 |
| 5 | *Proteus mirabilis* urinary tract infection (non-severe/outpatient), empirical therapy with ciprofloxacin, targeted therapy with trimethoprim-sulfamethoxazole. | from -45.51 to -24.51 |
| 6 | *Escherichia coli* ESBL severe urinary tract infection with sepsis, empirical therapy with piperacillin-tazobactam, targeted therapy with meropenem. | from +22.33 to +41.47 |
| 7 | *Streptococcus pyogenes* cellulitis in severe betalactam-allergy setting, empirical therapy with vancomycin, targeted therapy with levofloxacin. | from +14.13 to +26.25 |
| 8 | *Haemophilus influenzae* community-acquired pneumonia, empirical therapy with ampicillin, targeted therapy with co-amoxicillin. | from +12.46 to +23.14 |
| 9 | Methicillin-resistant coagulase-negative *Staphylococcus spp.* acute prosthetic joint infection, empirical therapy with co-amoxicillin, targeted therapy vancomycin and rifampin. | from +8.78 to +16.30 |
| 10 | Viridans streptococci native valve endocarditis, empirical therapy with co-amoxicillin, targeted therapy with ceftriaxone and gentamicin. | from +9.28 to +17.24 |

Examples of antimicrobial de-escalation events (ADE) for which the acceptable ΔS^3^ range was calculated based on the latest S^3^ database available (v14.0).
