# Supplementary material for: Simplified Spectrum Score (S3) app for pathogen-agnostic antimicrobial drug spectrum ranking to assess for antimicrobial de-escalation events
Source: Sci Rep. 2024 Apr 29;14:9776. doi: 10.1038/s41598-024-60041-6 (PMC11059348; doi:10.1038/s41598-024-60041-6)
Supplement: Supplementary file 7 — Supplementary Table S2. [file 41598_2024_60041_MOESM7_ESM.docx]

|  | **drug** | **v13** | **v14** | **average** | **std** | **CV% (std/average)** |
| --- | --- | --- | --- | --- | --- | --- |
| **0** | amikacin | 19.35 | 23.89 | 21.62 | 3.21 | 14.85 |
| **1** | amoxicillin | 15.41 | 13.38 | 14.40 | 1.44 | 9.98 |
| **2** | amoxicillin_clavulanate | 32.02 | 31.54 | 31.78 | 0.34 | 1.06 |
| **3** | ampicillin | 15.41 | 13.74 | 14.58 | 1.18 | 8.11 |
| **4** | ampicillin_sulbactam | 28.32 | 28.08 | 28.20 | 0.17 | 0.60 |
| **5** | azithromycin | 26.40 | 30.35 | 28.38 | 2.79 | 9.83 |
| **6** | aztreonam | 15.65 | 15.77 | 15.71 | 0.08 | 0.54 |
| **7** | cefazolin | 5.26 | 5.26 | 5.26 | 0.00 | 0.00 |
| **8** | cefepime | 45.16 | 44.44 | 44.80 | 0.51 | 1.13 |
| **9** | cefiderocol | 14.34 | 19.00 | 16.67 | 3.29 | 19.77 |
| **10** | cefotaxime | 32.38 | 32.74 | 32.56 | 0.25 | 0.78 |
| **11** | cefpodoxime | 14.34 | 13.74 | 14.04 | 0.42 | 3.01 |
| **12** | ceftaroline | 9.44 | 11.11 | 10.27 | 1.18 | 11.51 |
| **13** | ceftazidime | 32.38 | 33.45 | 32.92 | 0.76 | 2.31 |
| **14** | ceftazidime_avibactam | 38.95 | 38.83 | 38.89 | 0.08 | 0.22 |
| **15** | ceftobiprole | 6.81 | 16.37 | 11.59 | 6.76 | 58.32 |
| **16** | ceftolozane_tazobactam | 13.74 | 14.58 | 14.16 | 0.59 | 4.18 |
| **17** | ceftriaxone | 33.81 | 34.29 | 34.05 | 0.34 | 0.99 |
| **18** | cefuroxime | 9.56 | 9.44 | 9.50 | 0.08 | 0.89 |
| **20** | chloramphenicol | 20.43 | 20.79 | 20.61 | 0.25 | 1.23 |
| **21** | ciprofloxacin | 74.43 | 78.49 | 76.46 | 2.87 | 3.76 |
| **22** | clarithromycin | 7.41 | 11.11 | 9.26 | 2.62 | 28.28 |
| **23** | clindamycin | 52.69 | 52.57 | 52.63 | 0.08 | 0.16 |
| **24** | colistin | 11.71 | 11.47 | 11.59 | 0.17 | 1.46 |
| **25** | dalbavancin | 5.73 | 6.81 | 6.27 | 0.76 | 12.12 |
| **26** | daptomycin | 5.50 | 6.33 | 5.91 | 0.59 | 10.00 |
| **27** | delafloxacin | 59.38 | 59.26 | 59.32 | 0.08 | 0.14 |
| **28** | doxycycline | 50.78 | 48.27 | 49.52 | 1.77 | 3.58 |
| **29** | eravacycline | 8.24 | 20.79 | 14.52 | 8.87 | 61.11 |
| **30** | ertapenem | 50.54 | 49.22 | 49.88 | 0.93 | 1.86 |
| **31** | erythromycin | 20.79 | 24.13 | 22.46 | 2.37 | 10.53 |
| **32** | flucloxacillin | 2.87 | 2.87 | 2.87 | 0.00 | 0.00 |
| **33** | fosfomycin_iv | 17.56 | 18.16 | 17.86 | 0.42 | 2.36 |
| **34** | fosfomycin_po | 13.26 | 12.66 | 12.96 | 0.42 | 3.26 |
| **35** | fusidic_acid | 3.94 | 3.82 | 3.88 | 0.08 | 2.18 |
| **36** | gentamicin | 18.52 | 23.66 | 21.09 | 3.63 | 17.23 |
| **37** | imipenem | 68.22 | 89.84 | 79.03 | 15.29 | 19.35 |
| **38** | imipenem_relebactam | 70.73 | 90.32 | 80.53 | 13.85 | 17.21 |
| **39** | levofloxacin | 59.38 | 62.72 | 61.05 | 2.37 | 3.87 |
| **40** | linezolid | 39.55 | 43.13 | 41.34 | 2.53 | 6.13 |
| **41** | meropenem | 87.34 | 90.68 | 89.01 | 2.37 | 2.66 |
| **42** | meropenem_vaborbactam | 87.81 | 91.16 | 89.49 | 2.37 | 2.64 |
| **43** | metronidazole | 11.83 | 11.83 | 11.83 | 0.00 | 0.00 |
| **44** | minocycline | 7.29 | 7.29 | 7.29 | 0.00 | 0.00 |
| **45** | moxifloxacin | 42.41 | 42.89 | 42.65 | 0.34 | 0.79 |
| **46** | nitrofurantoin_po | 13.50 | 16.25 | 14.87 | 1.94 | 13.06 |
| **47** | none | 0.00 | 0.00 | 0.00 | 0.00 |  |
| **48** | oritavancin | 5.73 | 7.05 | 6.39 | 0.93 | 14.54 |
| **49** | oxacillin | 2.87 | 2.87 | 2.87 | 0.00 | 0.00 |
| **50** | penicillinG | 11.11 | 11.59 | 11.35 | 0.34 | 2.98 |
| **51** | penicillinV | 5.02 | 5.50 | 5.26 | 0.34 | 6.43 |
| **52** | piperacillin | 24.01 | 18.52 | 21.27 | 3.89 | 18.27 |
| **53** | piperacillin_tazobactam | 55.44 | 58.78 | 57.11 | 2.37 | 4.14 |
| **54** | quinupristin_dalfopristin | 4.90 | 4.18 | 4.54 | 0.51 | 11.16 |
| **55** | rifampicin | 29.51 | 29.51 | 29.51 | 0.00 | 0.00 |
| **56** | tedizolid | 25.33 | 43.13 | 34.23 | 12.59 | 36.77 |
| **57** | teicoplanin | 9.80 | 9.80 | 9.80 | 0.00 | 0.00 |
| **58** | tetracycline | 29.15 | 28.43 | 28.79 | 0.51 | 1.76 |
| **59** | ticarcillin_clavulanate | 18.28 | 18.28 | 18.28 | 0.00 | 0.00 |
| **60** | tigecycline | 23.30 | 23.30 | 23.30 | 0.00 | 0.00 |
| **61** | tobramycin | 17.80 | 22.70 | 20.25 | 3.46 | 17.10 |
| **62** | trimethoprim_sulfamethoxazole | 49.58 | 43.49 | 46.54 | 4.31 | 9.26 |
| **63** | vancomycin | 42.41 | 42.53 | 42.47 | 0.08 | 0.20 |
