# Supplementary material for: Simplified Spectrum Score (S3) app for pathogen-agnostic antimicrobial drug spectrum ranking to assess for antimicrobial de-escalation events
Source: Sci Rep. 2024 Apr 29;14:9776. doi: 10.1038/s41598-024-60041-6 (PMC11059348; doi:10.1038/s41598-024-60041-6)
Supplement: Supplementary file 8 — Supplementary Table S3. [file 41598_2024_60041_MOESM8_ESM.docx]

**Scenario: Acute gastroenteritis with bloodstream infection to *Salmonella spp.** adjudicated by Steward A (amoxicillin-clavulanate > ceftriaxone)**

|  |  |  | **West CH** | **S3 score ranking** | S-S-S-S-S | R-S-S-S-S | R-R-S-S-S | R-R-R-S-S | R-R-R-R-S | DOOR-MAT score |  | ceftriaxone (CTX) | co-amoxicillin (AMO-CLA) | azithromycin (AZT) | levofloxacin (LEVO) | meropenem (MER) |  |  |
| --- | --- | --- | --- | --- | --- | --- | --- | --- | --- | --- | --- | --- | --- | --- | --- | --- | --- | --- |
|  |  |  | Narrow | CTX | 1000 | 0 | 0 | 0 | 0 | 100 | **Ideal** | 100% | 0% | 0% | 0% | 0% |  |  |
|  |  |  | Intermediate I | AMO-CLA | 960 | 0 | 0 | 0 | 40 | 80 | **Slight OT** | 0% | 96% | 0% | 0% | 0% |  |  |
|  |  |  | Intermediate II | AZT | 940 | 0 | 0 | 0 | 60 | 60 | **Moderate OT** | 0% | 0% | 94% | 0% | 0% |  |  |
|  |  |  | Broad | LEVO | 820 | 0 | 0 | 0 | 180 | 40 | **Heavy OT** | 0% | 0% | 0% | 82% | 0% |  |  |
|  |  |  | Last Resort | MER | 1000 | 0 | 0 | 0 | 0 | 20 | **Severe OT** | 0% | 0% | 0% | 0% | 100% |  |  |
|  |  |  |  |  |  |  |  |  |  | 0 | **Inactive** | 0% | 4% | 6% | 18% | 0% |  |  |
|  |  |  |  |  |  |  |  |  |  |  |  | 100% | 100% | 100% | 100% | 100% |  |  |
|  |  |  |  |  |  |  |  |  |  |  |  |  |  |  |  |  |  |  |
|  |  |  |  |  |  |  |  |  |  |  |  |  |  |  |  |  |  |  |
|  |  |  |  |  | CTX | AMO-CLA | AZT | LEVO | MER |  |  |  |  |  |  |  |  |  |
|  |  |  |  | **Optimal** | 100% | 0% | 0% | 0% | 0% |  |  |  |  |  |  |  |  |  |
|  |  |  |  | **Appropriate** | 0% | 96% | 0% | 0% | 0% |  |  |  |  |  |  |  |  |  |
|  |  |  |  | **Inappropriate** | 0% | 0% | 94% | 82% | 100% |  |  |  |  |  |  |  |  |  |
|  |  |  |  | **Inactive** | 0% | 4% | 6% | 18% | 0% |  |  |  |  |  |  |  |  |  |
|  |  |  |  | DOOR-MAT average A (%) | 100 | 76.8 | 56.4 | 32.8 | 20 |  |  |  |  |  |  |  |  |  |

**** Epidemiological data from ANRESIS, Switzerland (***[***https://infect.swiss/***](https://infect.swiss/) ***simulating n=1000 for West Switzerland)***

**Scenario: Acute gastroenteritis with bloodstream infection to *Salmonella spp.** adjudicated by Steward A**

|  |  |  | **South CH** | **S3 score ranking** | S-S-S-S-S | R-S-S-S-S | R-R-S-S-S | R-R-R-S-S | R-R-R-R-S | DOOR-MAT score |  | ceftriaxone (CTX) | co-amoxicillin (AMO-CLA) | azithromycin (AZT) | levofloxacin (LEVO) | meropenem (MER) |  |  |
| --- | --- | --- | --- | --- | --- | --- | --- | --- | --- | --- | --- | --- | --- | --- | --- | --- | --- | --- |
|  |  |  | Narrow | CTX | 1000 | 0 | 0 | 0 | 0 | 100 | **Ideal** | 100% | 0% | 0% | 0% | 0% |  |  |
|  |  |  | Intermediate I | AMO-CLA | 710 | 0 | 0 | 0 | 290 | 80 | **Slight OT** | 0% | 71% | 0% | 0% | 0% |  |  |
|  |  |  | Intermediate II | AZT | 940 | 0 | 0 | 0 | 60 | 60 | **Moderate OT** | 0% | 0% | 94% | 0% | 0% |  |  |
|  |  |  | Broad | LEVO | 740 | 0 | 0 | 0 | 260 | 40 | **Heavy OT** | 0% | 0% | 0% | 74% | 0% |  |  |
|  |  |  | Last Resort | MER | 1000 | 0 | 0 | 0 | 0 | 20 | **Severe OT** | 0% | 0% | 0% | 0% | 100% |  |  |
|  |  |  |  |  |  |  |  |  |  | 0 | **Inactive** | 0% | 29% | 6% | 26% | 0% |  |  |
|  |  |  |  |  |  |  |  |  |  |  |  | 100% | 100% | 100% | 100% | 100% |  |  |
|  |  |  |  |  |  |  |  |  |  |  |  |  |  |  |  |  |  |  |
|  |  |  |  |  |  |  |  |  |  |  |  |  |  |  |  |  |  |  |
|  |  |  |  |  | CTX | AMO-CLA | AZT | LEVO | MER |  |  |  |  |  |  |  |  |  |
|  |  |  |  | **Optimal** | 100% | 0% | 0% | 0% | 0% |  |  |  |  |  |  |  |  |  |
|  |  |  |  | **Appropriate** | 0% | 71% | 0% | 0% | 0% |  |  |  |  |  |  |  |  |  |
|  |  |  |  | **Inappropriate** | 0% | 0% | 94% | 74% | 100% |  |  |  |  |  |  |  |  |  |
|  |  |  |  | **Inactive** | 0% | 29% | 6% | 26% | 0% |  |  |  |  |  |  |  |  |  |
|  |  |  |  | DOOR-MAT average A (%) | 100 | 56.8 | 56.4 | 29.6 | 20 |  |  |  |  |  |  |  |  |  |

**** Epidemiological data from ANRESIS, Switzerland (***[***https://infect.swiss/***](https://infect.swiss/) ***simulating n=1000 for South Switzerland)***

**Scenario: Acute gastroenteritis with bloodstream infection to *Salmonella spp.** adjudicated by Steward A**

|  |  |  | **Central East CH** | **S3 score ranking** | S-S-S-S-S | R-S-S-S-S | R-R-S-S-S | R-R-R-S-S | R-R-R-R-S | DOOR-MAT score |  | ceftriaxone (CTX) | co-amoxicillin (AMO-CLA) | azithromycin (AZT) | levofloxacin (LEVO) | meropenem (MER) |  |  |
| --- | --- | --- | --- | --- | --- | --- | --- | --- | --- | --- | --- | --- | --- | --- | --- | --- | --- | --- |
|  |  |  | Narrow | CTX | 1000 | 0 | 0 | 0 | 0 | 100 | **Ideal** | 100% | 0% | 0% | 0% | 0% |  |  |
|  |  |  | Intermediate I | AMO-CLA | 1000 | 0 | 0 | 0 | 0 | 80 | **Slight OT** | 0% | 100% | 0% | 0% | 0% |  |  |
|  |  |  | Intermediate II | AZT | 940 | 0 | 0 | 0 | 60 | 60 | **Moderate OT** | 0% | 0% | 94% | 0% | 0% |  |  |
|  |  |  | Broad | LEVO | 800 | 0 | 0 | 0 | 200 | 40 | **Heavy OT** | 0% | 0% | 0% | 80% | 0% |  |  |
|  |  |  | Last Resort | MER | 1000 | 0 | 0 | 0 | 0 | 20 | **Severe OT** | 0% | 0% | 0% | 0% | 100% |  |  |
|  |  |  |  |  |  |  |  |  |  | 0 | **Inactive** | 0% | 0% | 6% | 20% | 0% |  |  |
|  |  |  |  |  |  |  |  |  |  |  |  | 100% | 100% | 100% | 100% | 100% |  |  |
|  |  |  |  |  |  |  |  |  |  |  |  |  |  |  |  |  |  |  |
|  |  |  |  |  |  |  |  |  |  |  |  |  |  |  |  |  |  |  |
|  |  |  |  |  | CTX | AMO-CLA | AZT | LEVO | MER |  |  |  |  |  |  |  |  |  |
|  |  |  |  | **Optimal** | 100% | 0% | 0% | 0% | 0% |  |  |  |  |  |  |  |  |  |
|  |  |  |  | **Appropriate** | 0% | 100% | 0% | 0% | 0% |  |  |  |  |  |  |  |  |  |
|  |  |  |  | **Inappropriate** | 0% | 0% | 94% | 80% | 100% |  |  |  |  |  |  |  |  |  |
|  |  |  |  | **Inactive** | 0% | 0% | 6% | 20% | 0% |  |  |  |  |  |  |  |  |  |
|  |  |  |  | DOOR-MAT average A (%) | 100 | 80 | 56.4 | 32 | 20 |  |  |  |  |  |  |  |  |  |

**** Epidemiological data from ANRESIS, Switzerland (***[***https://infect.swiss/***](https://infect.swiss/) ***simulating n=1000 for Central East Switzerland)***

**Scenario: Acute gastroenteritis with bloodstream infection to *Salmonella spp.** adjudicated by Steward A**

|  |  |  | **Central West CH** | **S3 score ranking** | S-S-S-S-S | R-S-S-S-S | R-R-S-S-S | R-R-R-S-S | R-R-R-R-S | DOOR-MAT score |  | ceftriaxone (CTX) | co-amoxicillin (AMO-CLA) | azithromycin (AZT) | levofloxacin (LEVO) | meropenem (MER) |  |  |
| --- | --- | --- | --- | --- | --- | --- | --- | --- | --- | --- | --- | --- | --- | --- | --- | --- | --- | --- |
|  |  |  | Narrow | CTX | 960 | 0 | 0 | 0 | 40 | 100 | **Ideal** | 96% | 4% | 0% | 0% | 0% |  |  |
|  |  |  | Intermediate I | AMO-CLA | 960 | 40 | 0 | 0 | 40 | 80 | **Slight OT** | 0% | 96% | 0% | 0% | 0% |  |  |
|  |  |  | Intermediate II | AZT | 940 | 0 | 0 | 0 | 60 | 60 | **Moderate OT** | 0% | 0% | 94% | 0% | 0% |  |  |
|  |  |  | Broad | LEVO | 730 | 0 | 0 | 0 | 270 | 40 | **Heavy OT** | 0% | 0% | 0% | 73% | 0% |  |  |
|  |  |  | Last Resort | MER | 960 | 40 | 0 | 0 | 0 | 20 | **Severe OT** | 0% | 0% | 0% | 0% | 100% |  |  |
|  |  |  |  |  |  |  |  |  |  | 0 | **Inactive** | 4% | 0% | 6% | 27% | 0% |  |  |
|  |  |  |  |  |  |  |  |  |  |  |  | 100% | 100% | 100% | 100% | 100% |  |  |
|  |  |  |  |  |  |  |  |  |  |  |  |  |  |  |  |  |  |  |
|  |  |  |  |  |  |  |  |  |  |  |  |  |  |  |  |  |  |  |
|  |  |  |  |  | CTX | AMO-CLA | AZT | LEVO | MER |  |  |  |  |  |  |  |  |  |
|  |  |  |  | **Optimal** | 96% | 4% | 0% | 0% | 0% |  |  |  |  |  |  |  |  |  |
|  |  |  |  | **Appropriate** | 0% | 96% | 0% | 0% | 0% |  |  |  |  |  |  |  |  |  |
|  |  |  |  | **Inappropriate** | 0% | 0% | 94% | 73% | 100% |  |  |  |  |  |  |  |  |  |
|  |  |  |  | **Inactive** | 4% | 0% | 6% | 27% | 0% |  |  |  |  |  |  |  |  |  |
|  |  |  |  | DOOR-MAT average A (%) | 96 | 80.8 | 56.4 | 29.2 | 20 |  |  |  |  |  |  |  |  |  |

**** Epidemiological data from ANRESIS, Switzerland (***[***https://infect.swiss/***](https://infect.swiss/) ***simulating n=1000 for Central West Switzerland)***

**Scenario: Acute gastroenteritis with bloodstream infection to *Salmonella spp.** adjudicated by Steward A**

|  |  |  | **North West CH** | **S3 score ranking** | S-S-S-S-S | R-S-S-S-S | R-R-S-S-S | R-R-R-S-S | R-R-R-R-S | DOOR-MAT score |  | ceftriaxone (CTX) | co-amoxicillin (AMO-CLA) | azithromycin (AZT) | levofloxacin (LEVO) | meropenem (MER) |  |  |
| --- | --- | --- | --- | --- | --- | --- | --- | --- | --- | --- | --- | --- | --- | --- | --- | --- | --- | --- |
|  |  |  | Narrow | CTX | 1000 | 0 | 0 | 0 | 0 | 100 | **Ideal** | 100% | 0% | 0% | 0% | 0% |  |  |
|  |  |  | Intermediate I | AMO-CLA | 960 | 0 | 0 | 0 | 40 | 80 | **Slight OT** | 0% | 96% | 0% | 0% | 0% |  |  |
|  |  |  | Intermediate II | AZT | 940 | 0 | 0 | 0 | 60 | 60 | **Moderate OT** | 0% | 0% | 94% | 0% | 0% |  |  |
|  |  |  | Broad | LEVO | 1000 | 0 | 0 | 0 | 0 | 40 | **Heavy OT** | 0% | 0% | 0% | 100% | 0% |  |  |
|  |  |  | Last Resort | MER | 1000 | 0 | 0 | 0 | 0 | 20 | **Severe OT** | 0% | 0% | 0% | 0% | 100% |  |  |
|  |  |  |  |  |  |  |  |  |  | 0 | **Inactive** | 0% | 4% | 6% | 0% | 0% |  |  |
|  |  |  |  |  |  |  |  |  |  |  |  | 100% | 100% | 100% | 100% | 100% |  |  |
|  |  |  |  |  |  |  |  |  |  |  |  |  |  |  |  |  |  |  |
|  |  |  |  |  |  |  |  |  |  |  |  |  |  |  |  |  |  |  |
|  |  |  |  |  | CTX | AMO-CLA | AZT | LEVO | MER |  |  |  |  |  |  |  |  |  |
|  |  |  |  | **Optimal** | 100% | 0% | 0% | 0% | 0% |  |  |  |  |  |  |  |  |  |
|  |  |  |  | **Appropriate** | 0% | 96% | 0% | 0% | 0% |  |  |  |  |  |  |  |  |  |
|  |  |  |  | **Inappropriate** | 0% | 0% | 94% | 100% | 100% |  |  |  |  |  |  |  |  |  |
|  |  |  |  | **Inactive** | 0% | 4% | 6% | 0% | 0% |  |  |  |  |  |  |  |  |  |
|  |  |  |  | DOOR-MAT average A (%) | 100 | 76.8 | 56.4 | 40 | 20 |  |  |  |  |  |  |  |  |  |

**** Epidemiological data from ANRESIS, Switzerland (***[***https://infect.swiss/***](https://infect.swiss/) ***simulating n=1000 for North West Switzerland)***

**Scenario: Acute gastroenteritis with bloodstream infection to *Salmonella spp.** adjudicated by Steward A**

|  |  |  | **North East CH** | **S3 score ranking** | S-S-S-S-S | R-S-S-S-S | R-R-S-S-S | R-R-R-S-S | R-R-R-R-S | DOOR-MAT score |  | ceftriaxone (CTX) | co-amoxicillin (AMO-CLA) | azithromycin (AZT) | levofloxacin (LEVO) | meropenem (MER) |  |  |
| --- | --- | --- | --- | --- | --- | --- | --- | --- | --- | --- | --- | --- | --- | --- | --- | --- | --- | --- |
|  |  |  | Narrow | CTX | 970 | 0 | 0 | 0 | 30 | 100 | **Ideal** | 97% | 0% | 0% | 0% | 0% |  |  |
|  |  |  | Intermediate I | AMO-CLA | 920 | 0 | 0 | 0 | 80 | 80 | **Slight OT** | 0% | 92% | 0% | 0% | 0% |  |  |
|  |  |  | Intermediate II | AZT | 940 | 0 | 0 | 0 | 60 | 60 | **Moderate OT** | 0% | 0% | 94% | 0% | 0% |  |  |
|  |  |  | Broad | LEVO | 920 | 0 | 0 | 0 | 80 | 40 | **Heavy OT** | 0% | 0% | 0% | 92% | 0% |  |  |
|  |  |  | Last Resort | MER | 1000 | 0 | 0 | 0 | 0 | 20 | **Severe OT** | 0% | 0% | 0% | 0% | 100% |  |  |
|  |  |  |  |  |  |  |  |  |  | 0 | **Inactive** | 3% | 8% | 6% | 8% | 0% |  |  |
|  |  |  |  |  |  |  |  |  |  |  |  | 100% | 100% | 100% | 100% | 100% |  |  |
|  |  |  |  |  |  |  |  |  |  |  |  |  |  |  |  |  |  |  |
|  |  |  |  |  |  |  |  |  |  |  |  |  |  |  |  |  |  |  |
|  |  |  |  |  | CTX | AMO-CLA | AZT | LEVO | MER |  |  |  |  |  |  |  |  |  |
|  |  |  |  | **Optimal** | 97% | 0% | 0% | 0% | 0% |  |  |  |  |  |  |  |  |  |
|  |  |  |  | **Appropriate** | 0% | 92% | 0% | 0% | 0% |  |  |  |  |  |  |  |  |  |
|  |  |  |  | **Inappropriate** | 0% | 0% | 94% | 92% | 100% |  |  |  |  |  |  |  |  |  |
|  |  |  |  | **Inactive** | 3% | 8% | 6% | 8% | 0% |  |  |  |  |  |  |  |  |  |
|  |  |  |  | DOOR-MAT average A (%) | 97 | 73.6 | 56.4 | 36.8 | 20 |  |  |  |  |  |  |  |  |  |

**** Epidemiological data from ANRESIS, Switzerland (***[***https://infect.swiss/***](https://infect.swiss/) ***simulating n=1000 for North East Switzerland)***

**Scenario: Acute gastroenteritis with bloodstream infection to *Salmonella spp.** adjudicated by Steward B (amoxicillin-clavulanate < ceftriaxone)**

|  |  |  | **West CH** | **S^3^ score ranking** | S-S-S-S-S | R-S-S-S-S | R-R-S-S-S | R-R-R-S-S | R-R-R-R-S | DOOR-MAT score |  | co-amoxicillin (AMO-CLA) | ceftriaxone (CTX) | azithromycin (AZT) | levofloxacin (LEVO) | meropenem (MER) |  |  |
| --- | --- | --- | --- | --- | --- | --- | --- | --- | --- | --- | --- | --- | --- | --- | --- | --- | --- | --- |
|  |  |  | Narrow | AMO-CLA | 960 | 40 | 0 | 0 | 0 | 100 | **Ideal** | 96% | 4% | 0% | 0% | 0% |  |  |
|  |  |  | Intermediate I | CTX | 960 | 40 | 0 | 0 | 0 | 80 | **Slight OT** | 0% | 96% | 0% | 0% | 0% |  |  |
|  |  |  | Intermediate II | AZT | 940 | 0 | 0 | 0 | 60 | 60 | **Moderate OT** | 0% | 0% | 94% | 0% | 0% |  |  |
|  |  |  | Broad | LEVO | 820 | 0 | 0 | 0 | 180 | 40 | **Heavy OT** | 0% | 0% | 0% | 82% | 4% |  |  |
|  |  |  | Last Resort | MER | 960 | 40 | 0 | 0 | 0 | 20 | **Severe OT** | 0% | 0% | 0% | 0% | 96% |  |  |
|  |  |  |  |  |  |  |  |  |  | 0 | **Inactive** | 4% | 0% | 6% | 18% | 0% |  |  |
|  |  |  |  |  |  |  |  |  |  |  |  | 100% | 100% | 100% | 100% | 100% |  |  |
|  |  |  |  |  |  |  |  |  |  |  |  |  |  |  |  |  |  |  |
|  |  |  |  |  |  |  |  |  |  |  |  |  |  |  |  |  |  |  |
|  |  |  |  |  | AMO-CLA | CTX | AZT | LEVO | MER |  |  |  |  |  |  |  |  |  |
|  |  |  |  | **Optimal** | 96% | 4% | 0% | 0% | 0% |  |  |  |  |  |  |  |  |  |
|  |  |  |  | **Appropriate** | 0% | 96% | 0% | 0% | 0% |  |  |  |  |  |  |  |  |  |
|  |  |  |  | **Inappropriate** | 0% | 0% | 94% | 82% | 100% |  |  |  |  |  |  |  |  |  |
|  |  |  |  | **Inactive** | 4% | 0% | 6% | 18% | 0% |  |  |  |  |  |  |  |  |  |
|  |  |  |  | DOOR-MAT average A (%) | 96 | 80.8 | 56.4 | 32.8 | 20.8 |  |  |  |  |  |  |  |  |  |

**** Epidemiological data from ANRESIS, Switzerland (***[***https://infect.swiss/***](https://infect.swiss/) ***simulating n=1000 for West Switzerland)***

**Scenario: Acute gastroenteritis with bloodstream infection to *Salmonella spp.** adjudicated by Steward B**

|  |  |  | **South CH** | **S3 score ranking** | S-S-S-S-S | R-S-S-S-S | R-R-S-S-S | R-R-R-S-S | R-R-R-R-S | DOOR-MAT score |  | co-amoxicillin (AMO-CLA) | ceftriaxone (CTX) | azithromycin (AZT) | levofloxacin (LEVO) | meropenem (MER) |  |  |
| --- | --- | --- | --- | --- | --- | --- | --- | --- | --- | --- | --- | --- | --- | --- | --- | --- | --- | --- |
|  |  |  | Narrow | AMO-CLA | 710 | 290 | 0 | 0 | 0 | 100 | **Ideal** | 71% | 29% | 0% | 0% | 0% |  |  |
|  |  |  | Intermediate I | CTX | 710 | 290 | 0 | 0 | 0 | 80 | **Slight OT** | 0% | 71% | 0% | 0% | 0% |  |  |
|  |  |  | Intermediate II | AZT | 940 | 0 | 0 | 0 | 60 | 60 | **Moderate OT** | 0% | 0% | 94% | 21% | 0% |  |  |
|  |  |  | Broad | LEVO | 710 | 210 | 0 | 0 | 80 | 40 | **Heavy OT** | 0% | 0% | 0% | 71% | 29% |  |  |
|  |  |  | Last Resort | MER | 710 | 290 | 0 | 0 | 0 | 20 | **Severe OT** | 0% | 0% | 0% | 0% | 71% |  |  |
|  |  |  |  |  |  |  |  |  |  | 0 | **Inactive** | 29% | 0% | 6% | 8% | 0% |  |  |
|  |  |  |  |  |  |  |  |  |  |  |  | 100% | 100% | 0% | 100% | 100% |  |  |
|  |  |  |  |  |  |  |  |  |  |  |  |  |  |  |  |  |  |  |
|  |  |  |  |  |  |  |  |  |  |  |  |  |  |  |  |  |  |  |
|  |  |  |  |  | AMO-CLA | CTX | AZT | LEVO | MER |  |  |  |  |  |  |  |  |  |
|  |  |  |  | **Optimal** | 71% | 29% | 0% | 0% | 0% |  |  |  |  |  |  |  |  |  |
|  |  |  |  | **Appropriate** | 0% | 71% | 0% | 0% | 0% |  |  |  |  |  |  |  |  |  |
|  |  |  |  | **Inappropriate** | 0% | 0% | 0% | 92% | 100% |  |  |  |  |  |  |  |  |  |
|  |  |  |  | **Inactive** | 29% | 0% | 0% | 8% | 0% |  |  |  |  |  |  |  |  |  |
|  |  |  |  | DOOR-MAT average A (%) | 71 | 85.8 | 56.4 | 41 | 25.8 |  |  |  |  |  |  |  |  |  |

**** Epidemiological data from ANRESIS, Switzerland (***[***https://infect.swiss/***](https://infect.swiss/) ***simulating n=1000 for South Switzerland)***

**Scenario: Acute gastroenteritis with bloodstream infection to *Salmonella spp.** adjudicated by Steward B**

|  |  |  | **Central East CH** | **S3 score ranking** | S-S-S-S-S | R-S-S-S-S | R-R-S-S-S | R-R-R-S-S | R-R-R-R-S | DOOR-MAT score |  | co-amoxicillin (AMO-CLA) | ceftriaxone (CTX) | azithromycin (AZT) | levofloxacin (LEVO) | meropenem (MER) |  |  |
| --- | --- | --- | --- | --- | --- | --- | --- | --- | --- | --- | --- | --- | --- | --- | --- | --- | --- | --- |
|  |  |  | Narrow | AMO-CLA | 1000 | 0 | 0 | 0 | 0 | 100 | **Ideal** | 100% | 0% | 0% | 0% | 0% |  |  |
|  |  |  | Intermediate I | CTX | 1000 | 0 | 0 | 0 | 0 | 80 | **Slight OT** | 0% | 100% | 0% | 0% | 0% |  |  |
|  |  |  | Intermediate II | AZT | 940 | 0 | 0 | 0 | 60 | 60 | **Moderate OT** | 0% | 0% | 94% | 0% | 0% |  |  |
|  |  |  | Broad | LEVO | 800 | 0 | 0 | 0 | 200 | 40 | **Heavy OT** | 0% | 0% | 0% | 80% | 0% |  |  |
|  |  |  | Last Resort | MER | 1000 | 0 | 0 | 0 | 0 | 20 | **Severe OT** | 0% | 0% | 0% | 0% | 100% |  |  |
|  |  |  |  |  |  |  |  |  |  | 0 | **Inactive** | 0% | 0% | 6% | 20% | 0% |  |  |
|  |  |  |  |  |  |  |  |  |  |  |  | 100% | 100% | 100% | 100% | 100% |  |  |
|  |  |  |  |  |  |  |  |  |  |  |  |  |  |  |  |  |  |  |
|  |  |  |  |  |  |  |  |  |  |  |  |  |  |  |  |  |  |  |
|  |  |  |  |  | AMO-CLA | CTX | AZT | LEVO | MER |  |  |  |  |  |  |  |  |  |
|  |  |  |  | **Optimal** | 92% | 5% | 0% | 0% | 3% |  |  |  |  |  |  |  |  |  |
|  |  |  |  | **Appropriate** | 0% | 92% | 2% | 0% | 0% |  |  |  |  |  |  |  |  |  |
|  |  |  |  | **Inappropriate** | 0% | 0% | 92% | 92% | 97% |  |  |  |  |  |  |  |  |  |
|  |  |  |  | **Inactive** | 8% | 3% | 6% | 8% | 0% |  |  |  |  |  |  |  |  |  |
|  |  |  |  | DOOR-MAT average A (%) | 100 | 80 | 56.4 | 32 | 20 |  |  |  |  |  |  |  |  |  |

**** Epidemiological data from ANRESIS, Switzerland (***[***https://infect.swiss/***](https://infect.swiss/) ***simulating n=1000 for Central East Switzerland)***

**Scenario: Acute gastroenteritis with bloodstream infection to *Salmonella spp.** adjudicated by Steward B**

|  |  |  | **Central West CH** | **S3 score ranking** | S-S-S-S-S | R-S-S-S-S | R-R-S-S-S | R-R-R-S-S | R-R-R-R-S | DOOR-MAT score |  | co-amoxicillin (AMO-CLA) | ceftriaxone (CTX) | azithromycin (AZT) | levofloxacin (LEVO) | meropenem (MER) |  |  |
| --- | --- | --- | --- | --- | --- | --- | --- | --- | --- | --- | --- | --- | --- | --- | --- | --- | --- | --- |
|  |  |  | Narrow | AMO-CLA | 1000 | 0 | 0 | 0 | 0 | 100 | **Ideal** | 100% | 0% | 0% | 0% | 0% |  |  |
|  |  |  | Intermediate I | CTX | 960 | 0 | 0 | 0 | 40 | 80 | **Slight OT** | 0% | 96% | 0% | 0% | 0% |  |  |
|  |  |  | Intermediate II | AZT | 940 | 0 | 0 | 0 | 60 | 60 | **Moderate OT** | 0% | 0% | 94% | 0% | 0% |  |  |
|  |  |  | Broad | LEVO | 730 | 0 | 0 | 0 | 270 | 40 | **Heavy OT** | 0% | 0% | 0% | 73% | 0% |  |  |
|  |  |  | Last Resort | MER | 1000 | 0 | 0 | 0 | 0 | 20 | **Severe OT** | 0% | 0% | 0% | 0% | 100% |  |  |
|  |  |  |  |  |  |  |  |  |  | 0 | **Inactive** | 0% | 4% | 6% | 27% | 0% |  |  |
|  |  |  |  |  |  |  |  |  |  |  |  | 100% | 100% | 100% | 100% | 100% |  |  |
|  |  |  |  |  |  |  |  |  |  |  |  |  |  |  |  |  |  |  |
|  |  |  |  |  |  |  |  |  |  |  |  |  |  |  |  |  |  |  |
|  |  |  |  |  | AMO-CLA | CTX | AZT | LEVO | MER |  |  |  |  |  |  |  |  |  |
|  |  |  |  | **Optimal** | 92% | 5% | 0% | 0% | 3% |  |  |  |  |  |  |  |  |  |
|  |  |  |  | **Appropriate** | 0% | 92% | 2% | 0% | 0% |  |  |  |  |  |  |  |  |  |
|  |  |  |  | **Inappropriate** | 0% | 0% | 92% | 92% | 97% |  |  |  |  |  |  |  |  |  |
|  |  |  |  | **Inactive** | 8% | 3% | 6% | 8% | 0% |  |  |  |  |  |  |  |  |  |
|  |  |  |  | DOOR-MAT average A (%) | 100 | 76.8 | 56.4 | 29.2 | 20 |  |  |  |  |  |  |  |  |  |

**** Epidemiological data from ANRESIS, Switzerland (***[***https://infect.swiss/***](https://infect.swiss/) ***simulating n=1000 for Central West Switzerland)***

**Scenario: Acute gastroenteritis with bloodstream infection to *Salmonella spp.** adjudicated by Steward B**

|  |  |  | **North West CH** | **S3 score ranking** | S-S-S-S-S | R-S-S-S-S | R-R-S-S-S | R-R-R-S-S | R-R-R-R-S | DOOR-MAT score |  | co-amoxicillin (AMO-CLA) | ceftriaxone (CTX) | azithromycin (AZT) | levofloxacin (LEVO) | meropenem (MER) |  |  |
| --- | --- | --- | --- | --- | --- | --- | --- | --- | --- | --- | --- | --- | --- | --- | --- | --- | --- | --- |
|  |  |  | Narrow | AMO-CLA | 960 | 40 | 0 | 0 | 0 | 100 | **Ideal** | 96% | 4% | 0% | 0% | 0% |  |  |
|  |  |  | Intermediate I | CTX | 960 | 40 | 0 | 0 | 0 | 80 | **Slight OT** | 0% | 96% | 0% | 0% | 0% |  |  |
|  |  |  | Intermediate II | AZT | 940 | 0 | 0 | 0 | 60 | 60 | **Moderate OT** | 0% | 0% | 94% | 4% | 0% |  |  |
|  |  |  | Broad | LEVO | 960 | 40 | 0 | 0 | 0 | 40 | **Heavy OT** | 0% | 0% | 0% | 96% | 4% |  |  |
|  |  |  | Last Resort | MER | 960 | 40 | 0 | 0 | 0 | 20 | **Severe OT** | 0% | 0% | 0% | 0% | 96% |  |  |
|  |  |  |  |  |  |  |  |  |  | 0 | **Inactive** | 4% | 0% | 6% | 0% | 0% |  |  |
|  |  |  |  |  |  |  |  |  |  |  |  | 100% | 100% | 100% | 100% | 100% |  |  |
|  |  |  |  |  |  |  |  |  |  |  |  |  |  |  |  |  |  |  |
|  |  |  |  |  |  |  |  |  |  |  |  |  |  |  |  |  |  |  |
|  |  |  |  |  | AMO-CLA | CTX | AZT | LEVO | MER |  |  |  |  |  |  |  |  |  |
|  |  |  |  | **Optimal** | 92% | 5% | 0% | 0% | 3% |  |  |  |  |  |  |  |  |  |
|  |  |  |  | **Appropriate** | 0% | 92% | 2% | 0% | 0% |  |  |  |  |  |  |  |  |  |
|  |  |  |  | **Inappropriate** | 0% | 0% | 92% | 92% | 97% |  |  |  |  |  |  |  |  |  |
|  |  |  |  | **Inactive** | 8% | 3% | 6% | 8% | 0% |  |  |  |  |  |  |  |  |  |
|  |  |  |  | DOOR-MAT average A (%) | 96 | 80.8 | 56.4 | 40.8 | 20.8 |  |  |  |  |  |  |  |  |  |

**** Epidemiological data from ANRESIS, Switzerland (***[***https://infect.swiss/***](https://infect.swiss/) ***simulating n=1000 for North West Switzerland)***

**Scenario: Acute gastroenteritis with bloodstream infection to *Salmonella spp.** adjudicated by Steward B**

|  |  |  | **North East CH** | **S3 score ranking** | S-S-S-S-S | R-S-S-S-S | R-R-S-S-S | R-R-R-S-S | R-R-R-R-S | DOOR-MAT score |  | co-amoxicillin (AMO-CLA) | ceftriaxone (CTX) | azithromycin (AZT) | levofloxacin (LEVO) | meropenem (MER) |  |  |
| --- | --- | --- | --- | --- | --- | --- | --- | --- | --- | --- | --- | --- | --- | --- | --- | --- | --- | --- |
|  |  |  | Narrow | AMO-CLA | 920 | 0 | 0 | 0 | 80 | 100 | **Ideal** | 92% | 5% | 0% | 0% | 3% |  |  |
|  |  |  | Intermediate I | CTX | 920 | 50 | 0 | 0 | 30 | 80 | **Slight OT** | 0% | 92% | 2% | 0% | 0% |  |  |
|  |  |  | Intermediate II | AZT | 920 | 20 | 0 | 0 | 60 | 60 | **Moderate OT** | 0% | 0% | 92% | 0% | 0% |  |  |
|  |  |  | Broad | LEVO | 920 | 0 | 0 | 0 | 80 | 40 | **Heavy OT** | 0% | 0% | 0% | 92% | 5% |  |  |
|  |  |  | Last Resort | MER | 920 | 50 | 0 | 0 | 30 | 20 | **Severe OT** | 0% | 0% | 0% | 0% | 92% |  |  |
|  |  |  |  |  |  |  |  |  |  | 0 | **Inactive** | 8% | 3% | 6% | 8% | 0% |  |  |
|  |  |  |  |  |  |  |  |  |  |  |  | 100% | 100% | 100% | 100% | 100% |  |  |
|  |  |  |  |  |  |  |  |  |  |  |  |  |  |  |  |  |  |  |
|  |  |  |  |  |  |  |  |  |  |  |  |  |  |  |  |  |  |  |
|  |  |  |  |  | AMO-CLA | CTX | AZT | LEVO | MER |  |  |  |  |  |  |  |  |  |
|  |  |  |  | **Optimal** | 92% | 5% | 0% | 0% | 3% |  |  |  |  |  |  |  |  |  |
|  |  |  |  | **Appropriate** | 0% | 92% | 2% | 0% | 0% |  |  |  |  |  |  |  |  |  |
|  |  |  |  | **Inappropriate** | 0% | 0% | 92% | 92% | 97% |  |  |  |  |  |  |  |  |  |
|  |  |  |  | **Inactive** | 8% | 3% | 6% | 8% | 0% |  |  |  |  |  |  |  |  |  |
|  |  |  |  | DOOR-MAT average A (%) | 92 | 78.6 | 56.8 | 36.8 | 23.4 |  |  |  |  |  |  |  |  |  |

**** Epidemiological data from ANRESIS, Switzerland (***[***https://infect.swiss/***](https://infect.swiss/) ***simulating n=1000 for North East Switzerland)***
