# Supplementary material for: Simplified Spectrum Score (S3) app for pathogen-agnostic antimicrobial drug spectrum ranking to assess for antimicrobial de-escalation events
Source: Sci Rep. 2024 Apr 29;14:9776. doi: 10.1038/s41598-024-60041-6 (PMC11059348; doi:10.1038/s41598-024-60041-6)
Supplement: Supplementary file 2 — Supplementary Figure S1. [file 41598_2024_60041_MOESM2_ESM.pdf]

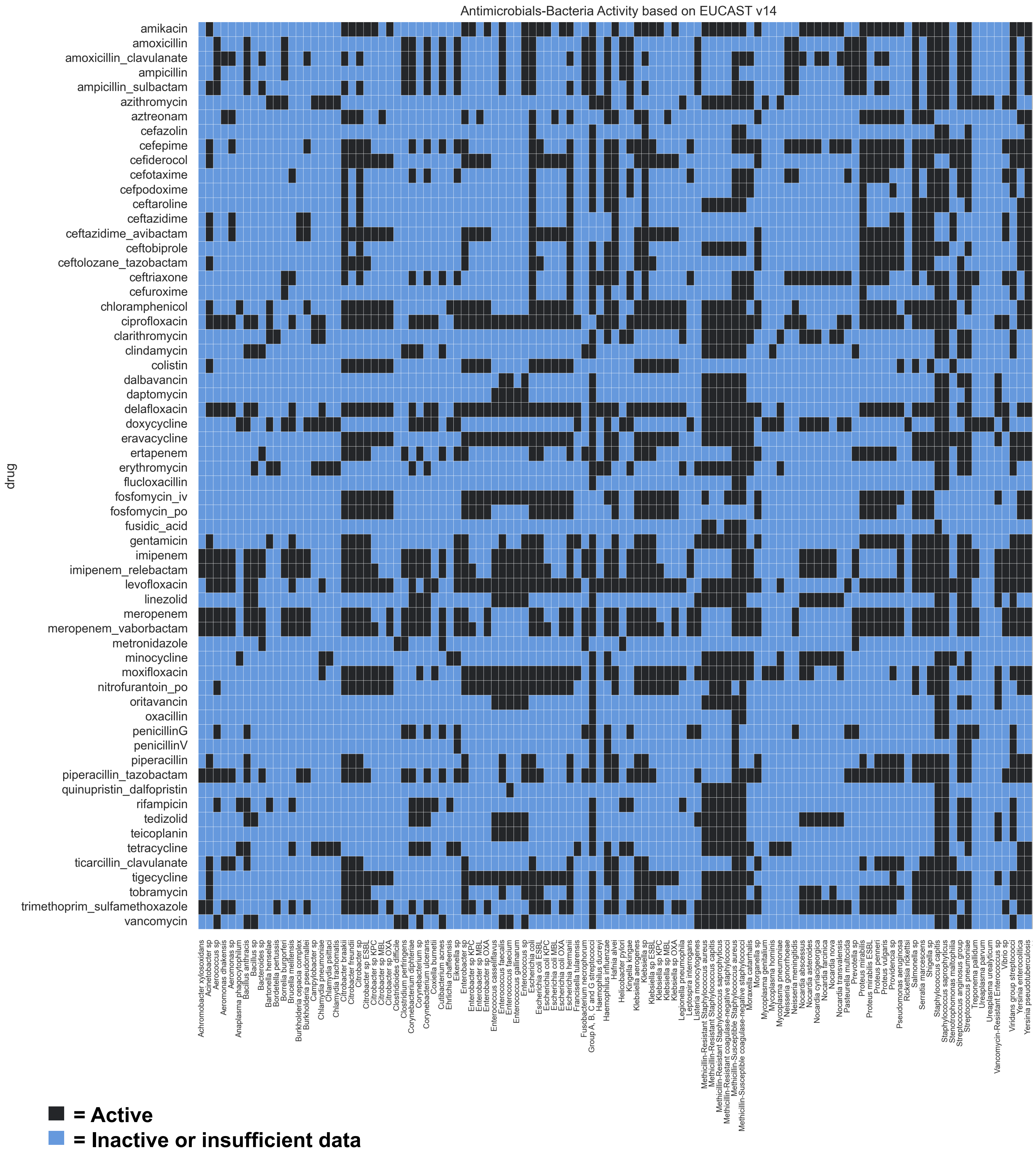
Antimicrobials-Bacteria Activity based on EUCAST v14
drug
amikacin
amoxicillin
amoxicillin_clavulanate
ampicillin
ampicillin_sulbactam
azithromycin
aztreonam
cefazolin
cefepime
cefiderocol
cefotaxime
cefpodoxime
ceftaroline
ceftazidime
ceftazidime_avibactam
ceftobiprole
ceftolozane_tazobactam
ceftriaxone
cefuroxime
chloramphenicol
ciprofloxacin
clarithromycin
clindamycin
colistin
dalbavancin
daptomycin
delafloxacin
doxycycline
eravacycline
ertapenem
erythromycin
flucloxacillin
fosfomycin_iv
fosfomycin_po
fusidic_acid
gentamicin
imipenem
imipenem_relebactam
levofloxacin
linezolid
meropenem
meropenem_vaborbactam
metronidazole
minocycline
moxifloxacin
nitrofurantoin_po
oritavancin
oxacillin
penicillinG
penicillinV
piperacillin
piperacillin_tazobactam
quinupristin_dalfopristin
rifampicin
tedizolid
teicoplanin
tetracycline
ticarcillin_clavulanate
tigecycline
tobramycin
trimethoprim_sulfamethoxazole
vancomycin
Achromobacter xylosoxidans
Acinetobacter sp
Aerococcus sp
Aeromonas dhakensis
Aeromonas sp
Anaplasma phagocytophilum
Bacillus anthracis
Bacillus sp
Bacteroides sp
Bartonella henselae
Bordetella pertussis
Borrelia burgdorferi
Brucella melitensis
Burkholderia cepacia complex
Burkholderia pseudomallei
Campylobacter sp
Chlamydia pneumoniae
Chlamydia psittaci
Chlamydia trachomatis
Citrobacter braakii
Citrobacter freundii
Citrobacter sp
Citrobacter sp ESBL
Citrobacter sp KPC
Citrobacter sp MBL
Citrobacter sp OXA
Clostridioides difficile
Clostridium perfringens
Corynebacterium diphteriae
Corynebacterium sp
Corynebacterium ulcerans
Coxiella burnetii
Cutibacterium acnes
Ehrlichia chaffeensis
Eikenella sp
Enterobacter sp
Enterobacter sp KPC
Enterobacter sp MBL
Enterobacter sp OXA
Enterococcus casseliflavus
Enterococcus faecalis
Enterococcus faecium
Enterococcus gallinarum
Enterococcus sp
Escherichia coli
Escherichia coli ESBL
Escherichia coli KPC
Escherichia coli MBL
Escherichia coli OXA
Escherichia hermannii
Francisella tularensis
Fusobacterium necrophorum
Group A, B, C and G streptococci
Haemophilus ducreyi
Haemophilus influenzae
Hafnia alvei
Helicobacter pylori
Kingella kingae
Klebsiella aerogenes
Klebsiella sp
Klebsiella sp ESBL
Klebsiella sp KPC
Klebsiella sp MBL
Klebsiella sp OXA
Legionella pneumophila
Leptospira interrogans
Listeria monocytogenes
Methicillin-Resistant Staphylococcus aureus
Methicillin-Resistant Staphylococcus capitis
Methicillin-Resistant Staphylococcus saprophyticus
Methicillin-Resistant coagulase-negative staphylococci
Methicillin-Susceptible Staphylococcus aureus
Methicillin-Susceptible coagulase-negative staphylococci
Moraxella catarrhalis
Morganella sp
Mycoplasma genitalium
Mycoplasma hominis
Mycoplasma pneumoniae
Neisseria gonorrhoeae
Neisseria meningitidis
Nocardia abscessus
Nocardia asteroides
Nocardia cyriacigeorgica
Nocardia farcinica
Nocardia nova
Nocardia transvalensis
Pasteurella multocida
Prevotella sp
Proteus mirabilis
Proteus mirabilis ESBL
Proteus penneri
Proteus vulgaris
Providencia sp
Pseudomonas aeruginosa
Rickettsia rickettsii
Salmonella sp
Serratia marcescens
Shigella sp
Staphylococcus capitis
Staphylococcus saprophyticus
Stenotrophomonas maltophilia
Streptococcus anginosus group
Streptococcus pneumoniae
Treponema pallidum
Ureaplasma parvum
Ureaplasma urealyticum
Vancomycin-Resistant Enterococcus sp
Vibrio sp
Viridans group streptococci
Yersinia enterocolitica
Yersinia pseudotuberculosis
= Active
= Inactive or insufficient data
